# Distinct roles of SOX9 in self-renewal of progenitors and mesenchymal transition of the endothelium

**DOI:** 10.1007/s10456-024-09927-7

**Published:** 2024-05-11

**Authors:** Jilai Zhao, Laura Sormani, Sebastien Jacquelin, Haiming Li, Cassandra Styke, Chenhao Zhou, Jonathan Beesley, Linus Oon, Simranpreet Kaur, Seen-Ling Sim, Ho Yi Wong, James Dight, Ghazaleh Hashemi, Abbas Shafiee, Edwige Roy, Jatin Patel, Kiarash Khosrotehrani

**Affiliations:** 1 Frazer Institute, The University of Queensland, Dermatology Research Centre, Experimental Dermatology Group, Brisbane, QLD 4102 Australia; 2 Mater Research, Translational Research Institute, Macrophage Biology Laboratory, Brisbane, QLD 4102 Australia; 3 Cancer Research Program, QIMR Berghofer Medical Research Institute, Brisbane, QLD 4006 Australia; 4 Centre for Ageing Research Program, Queensland University of Technology, Brisbane, QLD 4102 Australia

**Keywords:** Vessel-resident endothelial progenitors, Endothelial colony forming cells (ECFCs), Sox9, Endothelial-to-mesenchymal transition (EndMT), Oxidized low-density lipoprotein (oxLDL), High fat diet (HFD)

## Abstract

Regenerative capabilities of the endothelium rely on vessel-resident progenitors termed endothelial colony forming cells (ECFCs). This study aimed to investigate if these progenitors are impacted by conditions (i.e., obesity or atherosclerosis) characterized by increased serum levels of oxidized low-density lipoprotein (oxLDL), a known inducer of Endothelial-to-Mesenchymal Transition (EndMT). Our investigation focused on understanding the effects of EndMT on the self-renewal capabilities of progenitors and the associated molecular alterations. In the presence of oxLDL, ECFCs displayed classical features of EndMT, through reduced endothelial gene and protein expression, function as well as increased mesenchymal genes, contractility, and motility. Additionally, ECFCs displayed a dramatic loss in self-renewal capacity in the presence of oxLDL. RNA-sequencing analysis of ECFCs exposed to oxLDL validated gene expression changes suggesting EndMT and identified SOX9 as one of the highly differentially expressed genes. ATAC sequencing analysis identified SOX9 binding sites associated with regions of dynamic chromosome accessibility resulting from oxLDL exposure, further pointing to its importance. EndMT phenotype and gene expression changes induced by oxLDL in vitro or high fat diet (HFD) in vivo were reversed by the silencing of SOX9 in ECFCs or the endothelial-specific conditional knockout of Sox9 in murine models. Overall, our findings support that EndMT affects vessel-resident endothelial progenitor’s self-renewal. SOX9 activation is an early transcriptional event that drives the mesenchymal transition of endothelial progenitor cells. The identification of the molecular network driving EndMT in vessel-resident endothelial progenitors presents a new avenue in understanding and preventing a range of condition where this process is involved.

**Supplementary Information:**

The online version contains supplementary material available at 10.1007/s10456-024-09927-7.

## Introduction

Human endothelial colony forming cells (ECFCs), with the ability to self-renew in culture and form *de novo* vasculature, have been reported as a population of vessel-resident endothelial progenitors [1–6]. ECFCs can be isolated from various sources such as cord blood and human placental vasculature. Similarly, multiple teams have reported a population of murine endothelial cell with progenitor function as demonstrated by fate mapping and self-renewal assessment [2,7]. An important attribute of the endothelium is its ability to acquire mesenchymal traits in a process called endothelial-to-mesenchymal transition (EndMT). EndMT has been reported in physiological situations such as cardiac valve formation during fetal development as well as in pathological settings such as fibrotic diseases, atherosclerosis [8] and scarring such as wound healing [9,10]. Although many studies have evaluated the role of specific signalling pathways such as Notch or TGFbeta signalling, few have described the gene expression rewiring necessary for this transition and its impact on progenitor function.

Here, we aimed to elucidate molecular mechanisms involved in driving progenitor dysfunction and pathological mesenchymal trans-differentiation in a well-established model of EndMT relying on oxidised low-density lipoprotein (oxLDL) exposure in vitro and high fat diet (HFD) *in vivo.* We here show that EndMT results in endothelial progenitor dysfunction through significant chromosomal accessibility and transcriptional changes implicating SOX9 expression and activity. Additionally, the knockdown of *SOX9* in cultured ECFCs and murine model effectively abrogated the mesenchymal transition without altering self-renewal dysfunction highlighting the duality of function of *Sox9.*

## Methods

### Animals

All mice were treated in accordance with University of Queensland ethics approvals and guidelines for care of experimental animals under Animal Ethics Approval Certificate (AEC): UQCCR/472/18/NHMRC, UQCCR/473/NHMRC and UQDI/PACE/222/20. For endothelial specific knockout of the gene Sox9, Sox9fl-fl mice were crossed with Cdh5CreERt2/ROSAYfp to create the triple-transgenic Sox9fl/fl/ Cdh5CreERt2/ROSAYfp. Each mouse received a 2 mg dose of Tamoxifen (Sigma-Aldrich, MI, USA) per intraperitoneal injection.

### ECFCs isolation and culture

Human term placentas were obtained after informed written consent from healthy pregnant women and placental foetal ECFC isolated using our previously published protocol [11]. The use of human tissue was granted by the human ethics boards of The University of Queensland and the Royal Brisbane and Women’s Hospital. ECFCs were cultured on rat tail collagen coated tissue-culture flasks in Endothelial Growth Medium (EGM-2, Lonza Group, Basel, Switzerland) with 2% of foetal bovine serum (FBS).

### oxLDL treatment

ECFCs were cultured and treated with oxidized low-density lipoprotein sourced from human plasma (ox-LDL) (Invitrogen, # L34357) over a series of time points. The treatment duration spanned 3, 5, and 7 days, with an additional assessment conducted 5 days after transitioning to vehicle treatment for 2 days. At each time point, ECFCs were exposed to four concentrations of ox-LDL: 0 µg/ml (serving as the control or vehicle), 12.5 µg/ml, 25 µg/ml, and 50 µg/ml. The culture media was changed every 3 days during the treatment period. Following each time interval, ECFCs were harvested for further analysis.

### Inducible shSOX9

To generate inducible shRNA-expressing plasmids, the stuffer DNA was removed from Tet-pLKO-puro plasmid (RRID: Addgene_21915) by AgeI/EcoRI digest and replaced with double-stranded oligos encoding the SOX9-specific shRNA sequence obtained from the RNAi Consortium (TRCN0000352729, MISSION® TRC shRNA library, Sigma). Tet-pLKO-puro-Scrambled (shSCR) (RRID: Addgene_47541) was used as control.

### SOX9 overexpression

The SOX9 Lentiviral Vector (Human) (CMV) (pLenti-GIII-CMV) (ABM, #LV7-45154061) and its control Lenti-III-Blank vector (ABM, #LV587), were purchased from Applied Biological Materials.

Lentivirus were produced as previously described [12]. ECFCs (passage 4 to 6) were seeded on 6-well plates at a density of 2 × 10^5^ cells per well and incubated with virus for 72 h. Stably transduced ECFCs were selected by the addition of 1 µg/ml of puromycin (Sigma Aldrich, MO, CA) in culture media for 5 days.

### Self-renewal assay

A single-cell colony formation assay was utilised to assess ECFC self-renewal capacity. Treated and untreated ECFCs were sorted after Live/Dead discrimination via FACS into plates coated with 1% Collagen I and EGM2 + 10% FBS. Cells were allowed to proliferate with or without the addition of oxLDL. Brightfield images of the colony were taken after 14-days in culture, with the number of cells within each colony was counted and colonies classified as HPP (> 500 cells), LPP (> 250) colonies and an endothelial cluster (> 50). At least 100 single-cell colonies were counted per donor, per treatment, with a minimum of 5 donors per group.

### Matrigel capillary formation assay

100% Matrigel was plated onto ice-cold 96-well plates, before allowing for polymerisation at 37oC for 1 h. After which treated ECFCs were seeded onto the Matrigel and left to culture with or without oxLDL. 48-hours post seeding, images of capillaries were imaged on the Olympus iXplore Pro Inverted light microscope. Completed capillary networks were then counted on ImageJ.

### Collagen contraction assay

Treated ECFCs were resuspended in 3% type I bovine collagen solution at 1 × 10^6^ cells/mL. The cell/collagen mixture was then seeded into a 48-well plate and allowed to polymerise at 37°C for 1 h. After 24 h, the solidified collagen gel was gently lifted from the edge of the well. Photographs were taken at 24 h and 72 h post seeding, and the contracted area ratio was quantified using ImageJ.

### Scratch mobility assay

Treated ECFCs were seeded into 96-well plates at 5 × 10^4^ cells per well. 24 h post seeding, the scratch wound was achieved using an AutoScratch wound making Tool (BioTek, VT, USA) and EGM-2 supplemented with 10 µg/mL mitomycin C was then added. Images were then taken at 24- and 48-hours post wounding using the Olympus iXplore Pro Inverted light microscope and percentage scratch site migration was measured on ImageJ.

### Statistical analysis

All statistical analyses were performed using GraphPad Prism v9.0 software. Data were analyzed using the following tests: Mann Whitney (for non-parametric data), 1-way or 2-way ANOVA with multiple comparisons for parametric data. A p-value < 0.05 was considered significant. For all quantifications, data were averaged per a minimum of 4 biological replicates. Immunofluorescent and macroscopic quantifications were conducted with a minimum of 5 technical replicates per independent biological replicate.

## Results

### Exposure to oxLDL results in EndMT associated morphological changes and loss of self-renewal potential in ECFCs

**Fig. 1:**
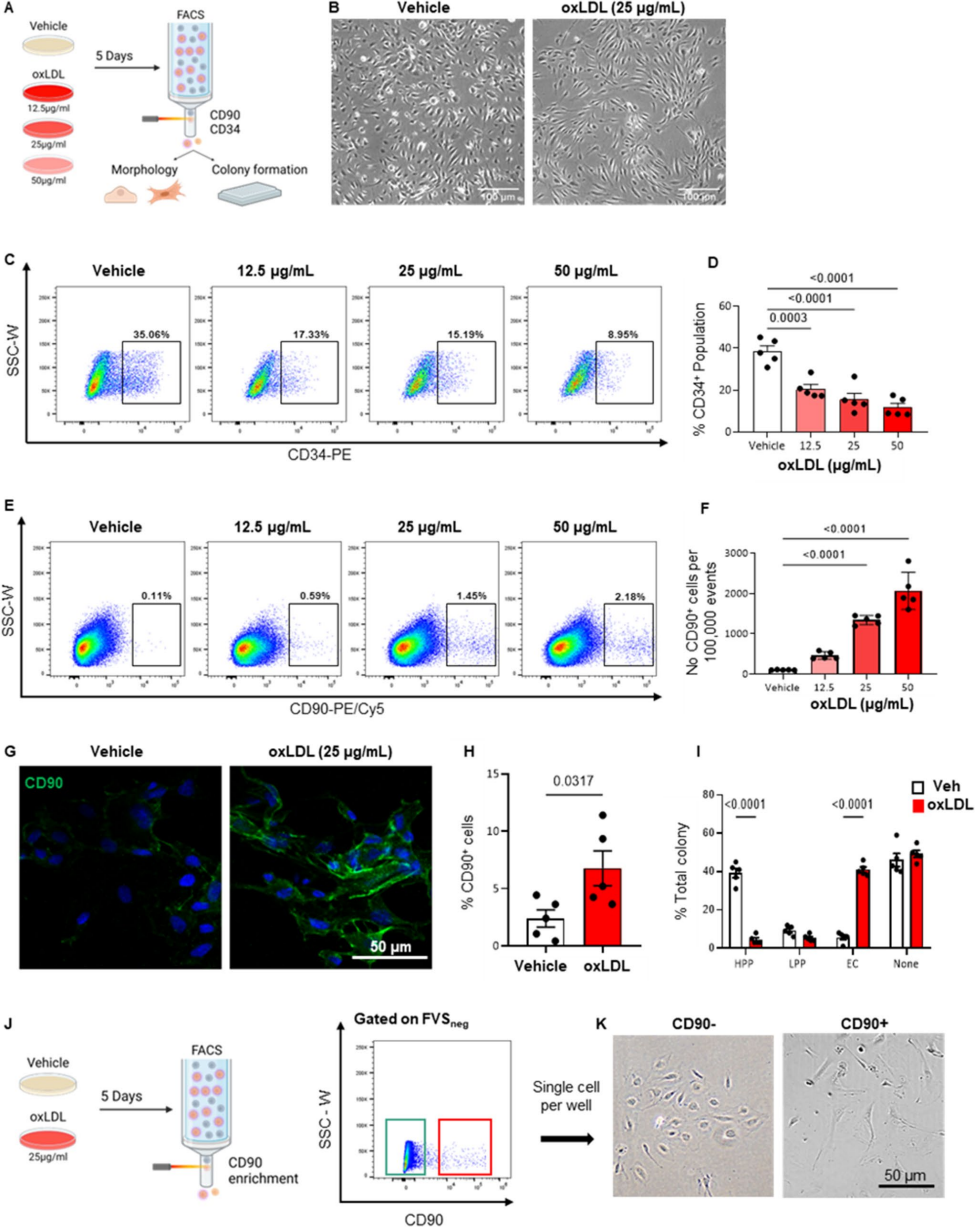
Exposure to oxLDL results in progenitor dysfunction and EndMT-associated changes in ECFCs. (**A**) Schematic representation of experimental timeline and design. ECFCs were treated with vehicle or oxLDL at 3 different concentrations (12.5, 25 and 50 µg/mL) for 5-days. Cellular changes were assessed by FACS and single-cell colony forming assay. (**B**) Morphological differences can be observed in culture after a 5-day oxLDL treatment period. (**C**-**F**) Flow cytometry analysis of endothelial marker CD34 and mesenchymal marker CD90 on oxLDL treated ECFCs. (vs. vehicle; *n* = 5 ECFCs isolated from biologically independent donors; mean ± SD; p value was calculated by two-way ANOVA with multiple comparison to Vehicle) (**G** and **H**) Immunofluorescent staining of CD90 on oxLDL treated ECFCs (vs. vehicle; *n* = 5 ECFCs isolated from biologically independent donors; mean ± SD; p value was calculated by one-way ANOVA with multiple comparison to Vehicle). (**I**) ECFCs were then FACS sorted at a single-cell level for the examination of proliferative potential (vs. vehicle; *n* = 5 ECFCs isolated from biologically independent donors; mean ± SD; p value was calculated by two-way ANOVA with multiple comparison to Vehicle) (**J** and **K**) Morphological assessment of CD90 + and CD90- oxLDL treated ECFCs single-cell sorted and cultured for 7 days without oxLDL

To model EndMT, we exposed ECFCs to oxLDL to study endothelial progenitors’ dysfunction and trans-differentiation [13] (Fig. 1A). Expectedly, an elongated and fusiform cell shape could be observed in sub-clusters of oxLDL treated ECFCs suggestive of EndMT (Fig. 1B). The changes in the expression of cell surface markers associated with oxLDL exposure were assessed by flow cytometry analysis. After 5 days of continuous oxLDL treatment, a significant and concentration-dependent decrease in the expression of CD34, a key endothelial marker, was noticed in ECFCs treated with oxLDL. Flow cytometry analysis showed 2.4 and 3.2-fold reduction in the percentage of CD34 + cells in ECFCs treated with 25 and 50 µg/mL of oxLDL respectively (*p* = 0.0001 vs. Veh, Fig. 1C and D). Additionally, CD90 (THY1), a key mesenchymal marker, was used to examine the gain of mesenchymal phenotype [14]. Conversely, 1.5 to 2.2% of ECFCs began to express CD90 at 25 and 50 µg/mL of oxLDL treatment respectively (*p* < 0.0001 vs. Veh, Fig. 1E and F). We confirmed this finding by immunostaining (Fig. 1G and H). Overall, these features validated the use of oxLDL as an established model of EndMT.

A single-cell colony formation assay was performed to determine the functional consequence of oxLDL treatment on ECFC self-renewal. ECFCs were treated with 25 µg/mL of oxLDL for 5 consecutive days and then sorted by FACS, plated 1 cell per well in endothelial cell growth medium-2 (EGM2) without oxLDL. Each flow-sorted cell was allowed to proliferate over 14 days. A systematic and prospective classification process was devised to categorize types of colonies formed by individual cells. A single-sorted ECFC is able to produce one of three types of colonies: a high proliferative potential (HPP) colony (> 500 cells at 14 days), a low proliferative potential (LPP) colony (> 250–500 cells), or an endothelial cluster (< 50 cells) [4]. Sorted ECFCs within the vehicle treatment group gave rise to a significant proportion (69 to 78% of the total number of wells containing cells) of HPP colonies versus the oxLDL treatment group, which comparatively demonstrated an 8-fold decrease (*p* < 0.0001 vs. Veh HPP, *n* = 5). Conversely, 71 to 86% of the colonies formed within the oxLDL group were determined as endothelial clusters, indicating a loss of self-renewal and proliferative capabilities during the 5-day exposure to oxLDL (*p* < 0.0001 vs. Veh EC, *n* = 5, Fig. 1I).

We noted a discordance in the percentage of ECFCs with reduced CD34 expression versus the small, although significant percentage of CD90 positives ECFCs suggestive of a transitional phenotype of ECFCs at an intermediate stage of trans-differentiation in the EndMT process. To evaluate the reversibility of the phenotype, we sorted oxLDL (25 µg/mL) treated ECFCs using CD90 marker and assessed their colony morphological changes in EGM2 without oxLDL at the single cell level (Fig. 1J). After 7 days, CD90 + ECFCs formed a mesenchymal spindle-shaped morphology. In contrast, CD90- colonies maintained a classical endothelial cobblestone-like morphology (Fig. 1K). However, following a 14-day culture in EGM2 medium, all cells regained an endothelial phenotype and exhibited a loss of CD90 surface marker expression (Supplemental Fig. 1A-C). This suggested a transition phase of reduced endothelial markers where endothelial morphology could be recovered and a more advanced mesenchymal phenotype with CD90 expression where the transition to mesenchymal phenotype was more pronounced that could also recover after longer time.

### EndMT dramatically alters chromatin accessibility and gene expression in ECFCs

**Fig. 2:**
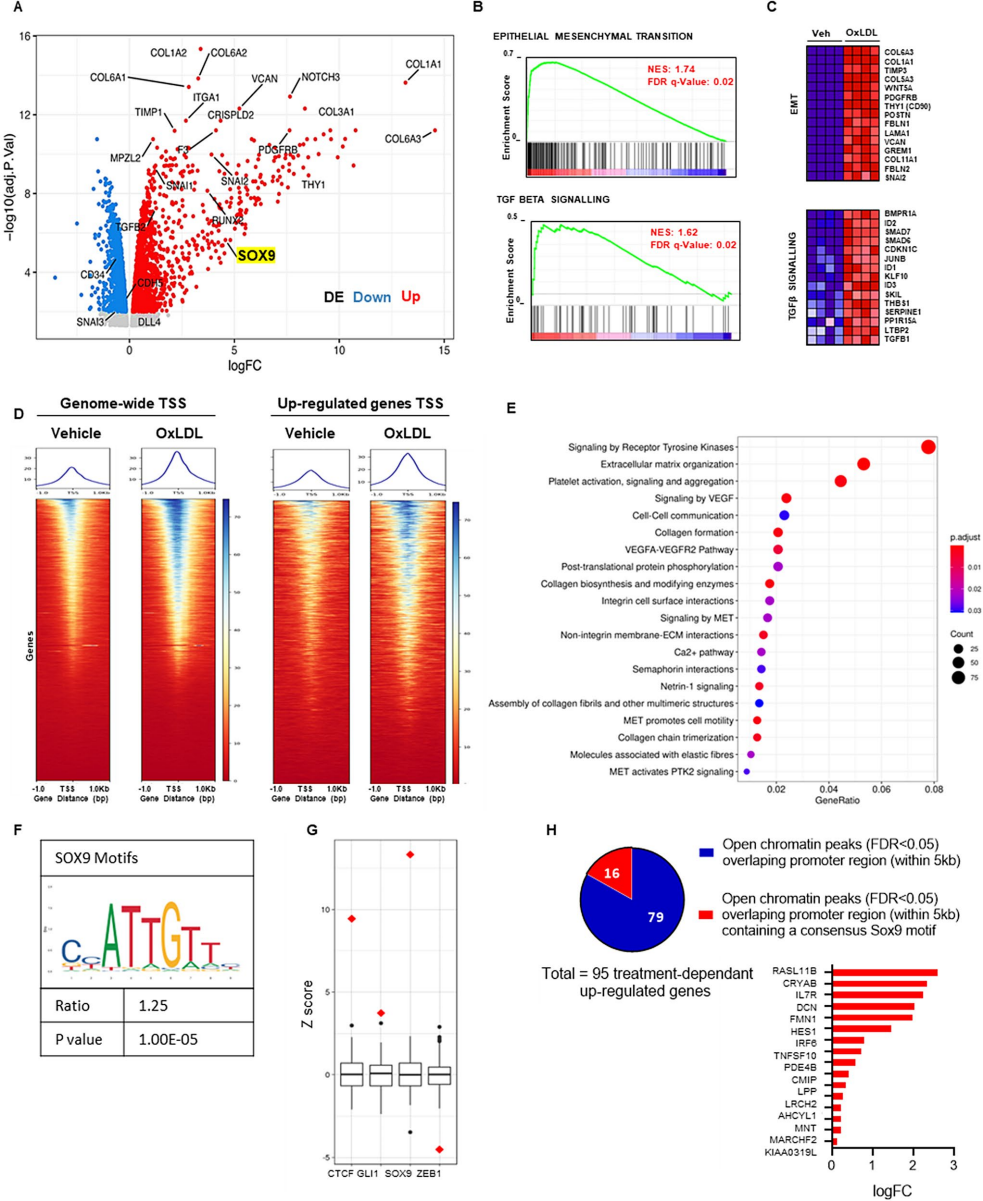
Bulk RNA and ATAC-sequencing analysis between vehicle and oxLDL-treated ECFC groups display a significant difference in gene expression and chromatin landscape. (**A**) Volcano plot of differentially expressed genes between vehicle and oxLDL (5-days, 25 µg/mL) treated ECFCs. *N* = 4 biological replicates per condition. Blue dots signify significantly down-regulated genes and red dots represents significantly up-regulated genes in oxLDL treated ECFCs compared to vehicle. (**B**) Gene Set Enrichment Analysis (GSEA) plot for oxLDL-enriched hallmark Epithelial to Mesenchymal Transition and TGFbeta signaling (Molecular Signatures Database, MSigDB) showing the profile of the running Enrichment Score (ES) and positions of gene set members on the rank ordered list. NES (top), and FDR q value (bottom rows) are indicated on GSEA plot. (**C**) Heatmap of the top 15 differentially expressed genes from the EMT and TGFbeta pathway gene sets. Expression values are represented as colours and range from red (high expression) to dark blue (lowest expression). (**D**) Heatmap profile of global enrichment for ATAC-seq peaks demonstrating chromatin accessibility at genome-wide transcription start sites (TSS) ± 1 kb and at up-regulated gene promoters ± 1 kb in vehicle and oxLDL treated ECFCs. The blue color intensity reflects the level of peak enrichment. Each condition has 4 biological replicates. (**E**) Pathway analysis for the differential ATAC-peaks (N up-regulated after oxLDL treatment at FDR < 0.05 = 3633). (**F**) HOMER analysis of SOX9 motifs enrichment. (**G**) Permutation-based strategy using ENCODE dNase-seq footprinting data. Motifs for selected TFs (SOX9, GLI1, ZEB1 and CTCF) were intersected with treatment-dependent open chromatin peaks. The observed number of intersected motifs (red diamonds) was compared to null background sets (boxplots). (**H**) Pie charts graph representing part of the whole number of open chromatin peaks overlapping promoter region (within 5 kb) of up-regulated genes in oxLDL treated ECFCs and containing one or more SOX9 motifs (red)

The established and irreversible mesenchymal phenotype and loss of self-renewal pointed towards epigenetic alterations, prompting us to evaluate gene expression and chromatin accessibility changes. We assessed transcriptomic changes of oxLDL treated ECFCs (25 µg/mL, 5-days) via bulk RNA sequencing (*n* = 4 ECFC from different donors per group). The oxLDL treatment resulted in the downregulation of 3128 genes and upregulation of 2554 genes (*p* < 0.05 after correction for multiple testing, Fig. 2A). Key attributes of the endothelium such as *CDH5, DLL4* and *CD34* expression were downregulated and the expression of classical mesenchymal genes such as *THY1*(CD90), *SNAI, RUNX2, BMP4* and *TGFβ2* as well as a large number of extracellular matrix genes were upregulated in ECFCs exposed to oxLDL. Several known molecular pathways involved in EndMT, including the epithelial to mesenchymal transition and TGF-beta signalling pathway were identified to be significantly altered by exposure to oxLDL [15–18]. This was further confirmed by GSEA (Gene Set Enrichment Analysis) showing over-representation of Epithelial to Mesenchymal Transition and TGFbeta signalling genes among those upregulated in ECFCs exposed to oxLDL (Fig. 2B and C). This analysis also revealed significant changes in expression of genes associated with other pathways such as hypoxia or TNFalpha signalling (Supplemental Fig. 2A and B). We identified *SOX9* among the top genes increased after oxLDL treatment (Fig. 2A), a chondrogenic/stem cell factor which has been previously reported to be expressed in murine endothelial progenitors, with its loss associated with endothelial differentiation as well as attenuated EndMT within the wound healing context in mice [10].

To assess the extent of oxLDL induced EndMT in ECFCs, we used a previously published bulk RNA sequencing dataset comparing human primary meso-endothelial bipotential placental cells that gave rise to both ECFCs and mesenchymal stem cells (MSC) in culture [19]. Additional transcriptional analysis was performed by cross comparison between oxLDL vs. Veh and ECFC precursors vs. MSC precursors datasets. A large proportion of differentially expressed genes (DEG) were shared between these two datasets (Supplemental Fig. 2C and D). Upon further pathway analysis of shared genes upregulated in ECFC precursors and downregulated in oxLDL treated ECFCs, we observed shared endothelial-specific pathways including “cell-cell adherent junction” and “PECAM1 interaction” strongly supporting the loss of endothelial phenotype of ECFC upon oxLDL exposure. Conversely, “collagen deposition”, “ECM organisation” and “TGF-beta signalling” pathways were shared between genes upregulated in MSC precursors and oxLDL treated ECFCs supporting the transition of ECFCs exposed to oxLDL towards a mesenchymal phenotype (Supplemental Fig. 2C and D). In addition, GSEA confirmed enrichment of fibroblast signatures and loss of fetal vascular endothelial cells signatures in the oxLDL treated cells (Supplemental Fig. 2E-H). Overall, gene expression changes occurring in ECFCs upon oxLDL exposure largely recapitulated features of EndMT.

We next asked if transcriptional changes in ECFCs associated with oxLDL driven EndMT were associated with modifications in chromatin accessibility and performed ATACseq analysis (*n* = 3 for oxLDL treatment and *n* = 4 for vehicle). The treatment by oxLDL led to significantly altered chromatin accessibility in ECFCs that clustered based on oxLDL treatment (Supplemental Fig. 3A and B). Peaks were distributed mostly around annotated gene promoters and introns (Supplemental Fig. 3C). As expected, ATAC-seq signal was enriched at transcriptional start sites (Supplemental Fig. 3D).

We identified 3633 significantly enriched peaks in the oxLDL compared to the vehicle samples (defined as FDR ≤ 0.05, and fold change > 1), and 30 regions that were more accessible in the vehicle compared to the treatment (fold change < -1). The analysis of the enrichment for ATAC-seq peaks at genome-wide regions surrounding transcription start sites (± 1 kb; TSS) showed that oxLDL treatment induced a global increase in chromatin accessibility (Fig. 2D). This list was narrowed down by focusing on genes with increased expression upon oxLDL exposure from our RNA-sequencing results, revealing a correlation in chromatin accessibility at these genes’ promoters. Looking at enriched pathways after treatment among the differential ATAC-peaks (N up-regulated at FDR < 0.05 = 3633) and assuming that the nearest gene to the peak is a target gene, we could identify “extracellular matrix organisation” and “collagen formation” that were reminiscent of the “EMT pathways” enriched in RNAseq results (Fig. 2E). Interestingly, key signalling pathways associated with endothelial cells were also identified such as VEGF signalling. This highlights that ECFCs undergoing EndMT maintain an open chromatin allowing the potential re-expression of endothelial genes as observed in the CD90- fraction of ECFCs.

We next looked at transcription factor binding motifs in oxLDL treatment-dependent open chromatin. HOMER analysis showed that peaks upregulated upon oxLDL exposure were enriched for binding motifs of AP1-cJUN and CTCF (Supplemental Fig. 3G). We then asked if the changes observed in open chromatin could reflect classical transcription factors involved in EndMT. We compared SOX9, CTCF, GLI1 or ZEB1 motifs in the oxLDL dependent open chromatin using permutation analysis to reveal a greater overrepresentation of SOX9 motifs as compared to other transcription factors (Fig. 2G). Indeed, motifs associated with SOX9 activity (Fig. 2F) demonstrated a 1.25-fold increase in oxLDL-dependent open chromatin regions. Among the treatment-dependent peaks (*N* = 3633), 847 (23%) contained one or more SOX9 motif. This was 1.7-fold greater than what would be expected by chance, based on a random sampling strategy.

When examining genes with upregulated expression identified by RNAseq in oxLDL treatment, 95 were found to have an oxLDL-dependent open chromatin peak in proximity to their promoter region (within 5 kb), and 16 of those genes contained a SOX9 binding motif (Fig. 2H). This possibly reflects key genes where SOX9 is more directly responsible for the overexpression. Twelve of them (*RASL11B*, *CRYAB*, *IL7R*, *DCN*, *FMN1*, *HES1*, *IRF6*, *TNFSF10*, *PDE4B*, *LRCH2*, *AHCYL1* and *MNT*) are associated with Atherosclerosis (a condition where EndMT is known to play a role) according to the CTD Gene-Disease Associations dataset and the gene set enrichment analysis (Supplemental Fig. 3E). Finally, vascular smooth muscle and myofibroblasts cell type signatures were found to be associated with these 16 genes containing a SOX9 binding motif (Supplemental Fig. 3F).

Together, these observations demonstrate that in vitro exposure of ECFCs to oxLDL increases chromatin accessibility leading to the expression of a transcriptional program allowing mesenchymal transition associated with reduced expression of endothelial genes. Among transcription factors potentially driving these chromatin accessibility changes, SOX9 could play an important role given the over-representation of its binding domains.

**Fig. 3:**
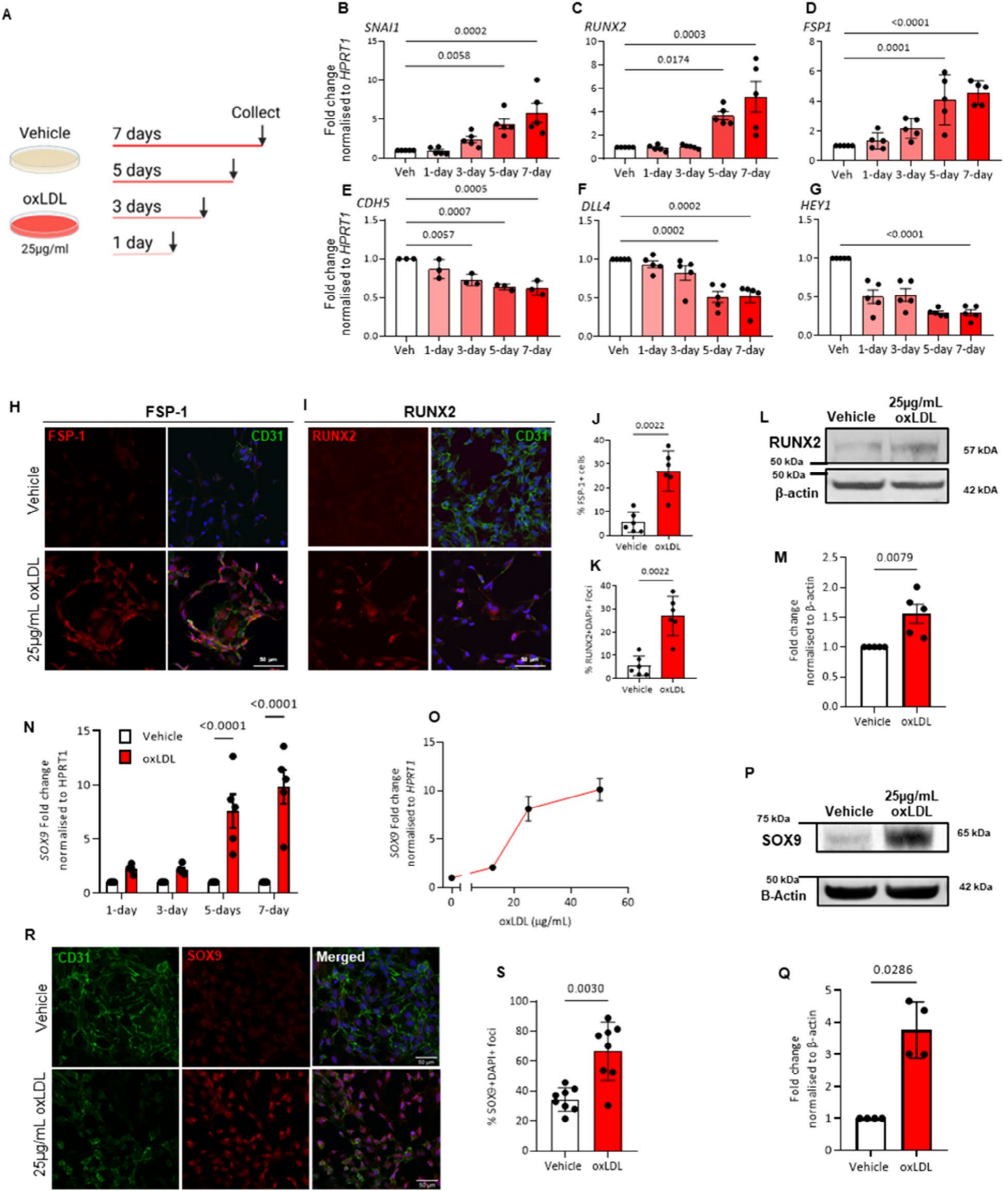
oxLDL-treated ECFCs lose the expression of endothelial cell-specific genes and initiate the expression of mesenchymal cell-specific genes. (**A**) Schematic representation of treatment timeline and oxLDL dose. (**B**-**G**) mRNA expression of mesenchymal and endothelial genes after 25 µg/mL oxLDL treatment at varied timepoints. (vs. respective timepoint vehicle; *n* = 5; 5 ECFCs isolated from biologically independent donors; mean ± SD; p value was calculated by one-way ANOVA with multiple comparison to Vehicle). (**H**-**K**) Immunofluorescent quantification of FSP-1 and RUNX2 protein expression in ECFCs exposed to 25 µg/mL oxLDL for 5 days (vs. vehicle; *n* = 5; 5 ECFCs isolated from biologically independent donors; mean ± SD; p value was calculated by Mann–Whitney U test to Vehicle). (**L** and **M**) Western blot analysis of RUNX2 expression in ECFCs treated with 25 µg/mL (**N** and **O**) SOX9 mRNA expression in ECFCs at specific timepoints of oxLDL exposure and oxLDL concentrations. (**P**-**S**) Immunofluorescent quantification and western blot analysis of SOX9 expression in ECFCs treated with 25 µg/mL oxLDL for 5 days (vs. vehicle; *n* = 5; 5 ECFCs isolated from biologically independent donors; mean ± SD; p value was calculated by Mann–Whitney U test to Vehicle)

To further understand and validate the molecular kinetics of oxLDL treatment and associated EndMT changes in ECFCs, the key genes identified from the RNA-seq were examined at various timepoints at mRNA and protein levels (Fig. 3A). Firstly, *SOX9* mRNA expression levels were assessed at various timepoints and oxLDL concentrations compared to respective time-matched vehicle control. Exposure to 25 µg/mL oxLDL resulted in the significant increase of *SOX9* expression only by day 5 (*p* < 0.0001 vs. Veh 5-days, Fig. 3N). Immunoblotting and immunofluorescent staining of SOX9 further supported this increase at the protein level (*p* = 0.0030 vs. Veh, *p* = 0.0286 vs. Veh respectively, Fig. 3P-S). oxLDL treatment (25 µg/mL) resulted in a 3 to 6-fold increase in the expression of mesenchymal markers *SNAI1* and *FSP-1*, as well as osteogenic marker *RUNX2* (*p* < 0.0174 vs. Veh, Fig. 3B-D), with increasing expression occurring mostly from D5 of exposure to oxLDL treatment mirroring the expression of *SOX9*. Consistently, when using immunostaining, FSP-1 and RUNX2 were also expressed at higher levels in oxLDL treated ECFCs compared to vehicle group (*p* = 0.0022 vs. Veh, Fig. 3H-K). The observed increase of RUNX2 expression at protein level was confirmed by Western Blot (*p* = 0.0079 vs. Veh, Fig. 3L and M). Furthermore, the expression of the endothelial adhesion gene, *CDH5* and those involved in the Notch signalling pathway (*DLL4, HEY1*) were significantly reduced in the oxLDL treated group (*p* < 0.007 vs. Veh, Fig. 3E-G). However, in the case of these genes the changes in expression occurred more continuously over time and before D5.

### *SOX9 shRNA* knockdown attenuates oxLDL induced progenitor dysfunction and EndMT whereas *SOX9* overexpression mimics EndMT

**Fig. 4:**
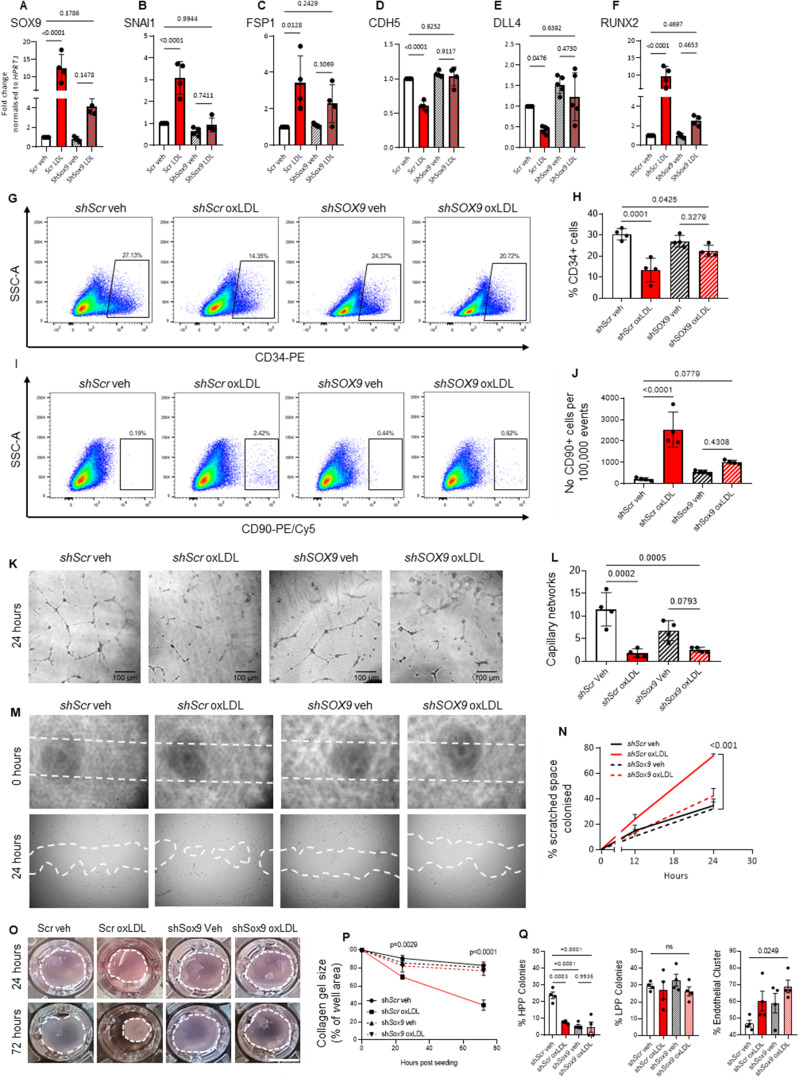
SOX9 shRNA silencing attenuates oxLDL-induced progenitor dysfunction and EndMT. (**A**-**F**) Mesenchymal and endothelial mRNA expression assessed by qPCR. (**G**-**J**) FACS analysis of endothelial marker CD34 and mesenchymal marker CD90 expression of ECFCshScr and ECFCshSox9 exposed to 25 µg/mL oxLDL. (**K** and **L**) Capillary tube formation assessment of ECFCshScr and ECFCshSox9 exposed to 25 µg/mL oxLDL. (**M** and **N**) Analysis of ECFC motility by scratch assay. Quantification of the scratched area invaded by ECFCshScr and ECFCshSox9 treated with vehicle or oxLDL for 5 day within a 24-hour time-lapse (**O** and **P**) 3D collagen matrix gel contraction assay of ECFCshScr and ECFCshSox9 treated with vehicle or oxLDL, images taken at 24 h and 72 h post seeding. (**Q**) ECFC proliferative capability was then assessed by single-cell colony formation assay. (*n* = 4; 2 biologically independent ECFC donors, from 2 independent experiments; mean ± SD; p value was calculated by one-way ANOVA with multiple comparison)

To further investigate the role of SOX9 in oxLDL-induced progenitor dysfunction and EndMT, transduced ECFCs expressing an shRNA against *SOX9* upon tetracycline induction were generated from 2 different donors, here termed ECFC [9]. Integration and expression of *shSOX9* was first validated using immunoblotting of the TetR repressor (Supplementary Fig. 4A). Also, the impact on *SOX9* expression was measured using a various dose of tetracyclin demonstrating about 50% reduction at protein and RNA level (Supplementary Fig. 4B and C). Our data showed that EndMT-associated genes identified within the RNA sequencing to be regulated by oxLDL treatment *SNAI1, FSP-1, CDH5* and *DLL4* were not affected by the knockdown of *SOX9* at baseline in the absence of oxLDL (Fig. 4A-F). Interestingly, within the vehicle treated group, the expression of *SOX9* was not affected by the knockdown, consistent with its low/negligible expression at the basal level. *SOX9* expression was induced (15-fold increased) upon oxLDL exposure (5 days, 25 µg/mL, *p* < 0.0001). Upon silencing, the increase in *SOX9* expression after oxLDL exposure was reduced (4-fold only) and not significantly different from vehicle, suggesting effective blockade of *SOX9* expression by the shRNA. Strikingly, after oxLDL exposure, *SOX9* silencing was associated with a near-complete restoration of expression of endothelial genes (*CDH5, DLL4*) significantly reduced in the ECFC^*shScr*^. Conversely, the silencing of SOX9 strongly inhibited the aberrant expression of other mesenchymal genes induced by oxLDL treatment such as *SNAI1* and *RUNX2* and a partial but significant (66% reduction) attenuation for *FSP1* within ECFC [9] compared to control ECFC^*shScr*^ (Fig. 4A-F). In parallel, the flow cytometry analysis for CD34 and CD90 proteins was performed to determine whether this change in gene expression could influence the expression of key CD markers. While the CD34 + population was reduced upon oxLDL treatment, this was only partly restored by *SOX9* silencing (*p* = 0.0425, Fig. 4 G and H). In contrast, the increased number of CD90 + cells after oxLDL treatment was effectively reversed in the ECFC [9] group (Fig. 4 I and J). These lines of evidence appear to be indicative of an attenuation of oxLDL-induced EndMT upon *SOX9* silencing.

Given these changes in gene expression, we next examined if *SOX9* knockdown could affect EndMT at the functional level. Initial assessment through Matrigel capillary formation and single cell colony formation assay demonstrated a significant loss of endothelial-related functions after oxLDL treatment (*p* < 0.001 vs. ECFC^*shScr*^ Veh, Fig. 4K-P). Unexpectedly, neither the capillary nor single cell colony formation capabilities of ECFCs were recovered by *SOX9* silencing after oxLDL treatment. Indeed, the silencing of *SOX9* alone in ECFCs in the absence of oxLDL exposure was sufficient in directly and significantly inhibiting ECFC self-renewal, with decrease in the percentage of HPP colonies formed comparable to oxLDL treated ECFCs (*p* < 0.001 vs. ECFC [9] Veh, Fig. 4Q). The low basal expression of *SOX9* may therefore be needed to maintain the ECFC progenitor population. Upon shRNA silencing of SOX9, oxLDL did not further affect the endothelial function of ECFCs in terms of capillary formation (*p* = 0.0793) or HPP colony formation (*p* = 0.99) as compared to ECFC [9] exposed to vehicle alone.

To investigate the role of SOX9 on the gain of mesenchymal attributes upon oxLDL exposure, scratch mobility and 3D collagen gel contraction assays were used. As part of EndMT activation, endothelial cells display enhanced migratory functions, reflected here, with oxLDL treated ECFC^*shScr*^ colonizing significantly higher percentage of scratched space compared to vehicle (*p* < 0.001 vs. ECFC^*shScr*^ Veh, Fig. 4M and N). This increased mobility was abrogated within the ECFC [9] group. Furthermore, the gain of specific-mesenchymal function can also be demonstrated through the significant increase in cell contractility with more than 50% gel size reduction after 72 h (*p* < 0.001 vs. ECFC^*shScr*^ oxLDL, Fig. 4O and P). This process is generally attributed to fibroblasts differentiating into a more contractile state as myofibroblast. This transition is also supported at the molecular level, as an array of myofibroblast specific genes (*ACTA2, COL1A1, COL1A2, TGFB*) were found to be significantly up regulated within the RNAseq after oxLDL treatment. Equally, this functional transition was efficiently reversed in ECFC [9] treated with oxLDL, demonstrating a central role of SOX9 in driving oxLDL-induced EndMT at both the functional and molecular levels. These findings corroborate that the silencing of *SOX9* can effectively attenuate mesenchymal transition by inhibiting the aberrant expression of key drivers of this process (*SNAI1, SLUG*). However, it was not able to restore endothelial and progenitor function.

To further establish the importance of SOX9 in the EndMT phenotype, we next proceeded with its overexpression. ECFCs overexpressing SOX9 (Ov*SOX9*) were compared to ECFCs harbouring only the empty vector (EV). As a result of the overexpression, SOX9 levels were more than 5000-fold increased in ECFC OvSOX^9^ (Supplemental Fig. 5A). This increased expression led to morphological alterations in the absence of oxLDL along with elevated expression levels of mesenchymal genes such as *COL2A1* and *SNAI1 or* CD90 at the protein level (Supplemental Fig. 5B-4E). Functionally, overexpression of SOX9 dramatically reduced HPP colony forming potential resulting in increasing numbers of endothelial clusters pointing to a loss of self-renewal (Supplemental Fig. 5D). Similarly, SOX9 overexpression resulted in a reduction in tube formation (Supplemental Fig. 5F, G). Overall, SOX9 overexpression recapitulated gene expression and endothelial functional changes observed in EndMT induced by oxLDL.

### Endothelial-specific ablation of *Sox9* attenuates EndMT in vivo

We next examined if our observations in ECFCs could be reproduced in vivo. To examine this, *Cdh5Cre*^*ERt2*^*/ROSAYfp/Sox9*^*+/+*^ (*Sox9*^*eWT*^) and *Cdh5Cre*^*ERt2*^*/ROSAYfp/Sox9*^*flox/flox*^ (*Sox9*^*eKO*^) mice were generated to examine the impact of endothelial-specific deletion of *Sox9*.

**Fig. 5:**
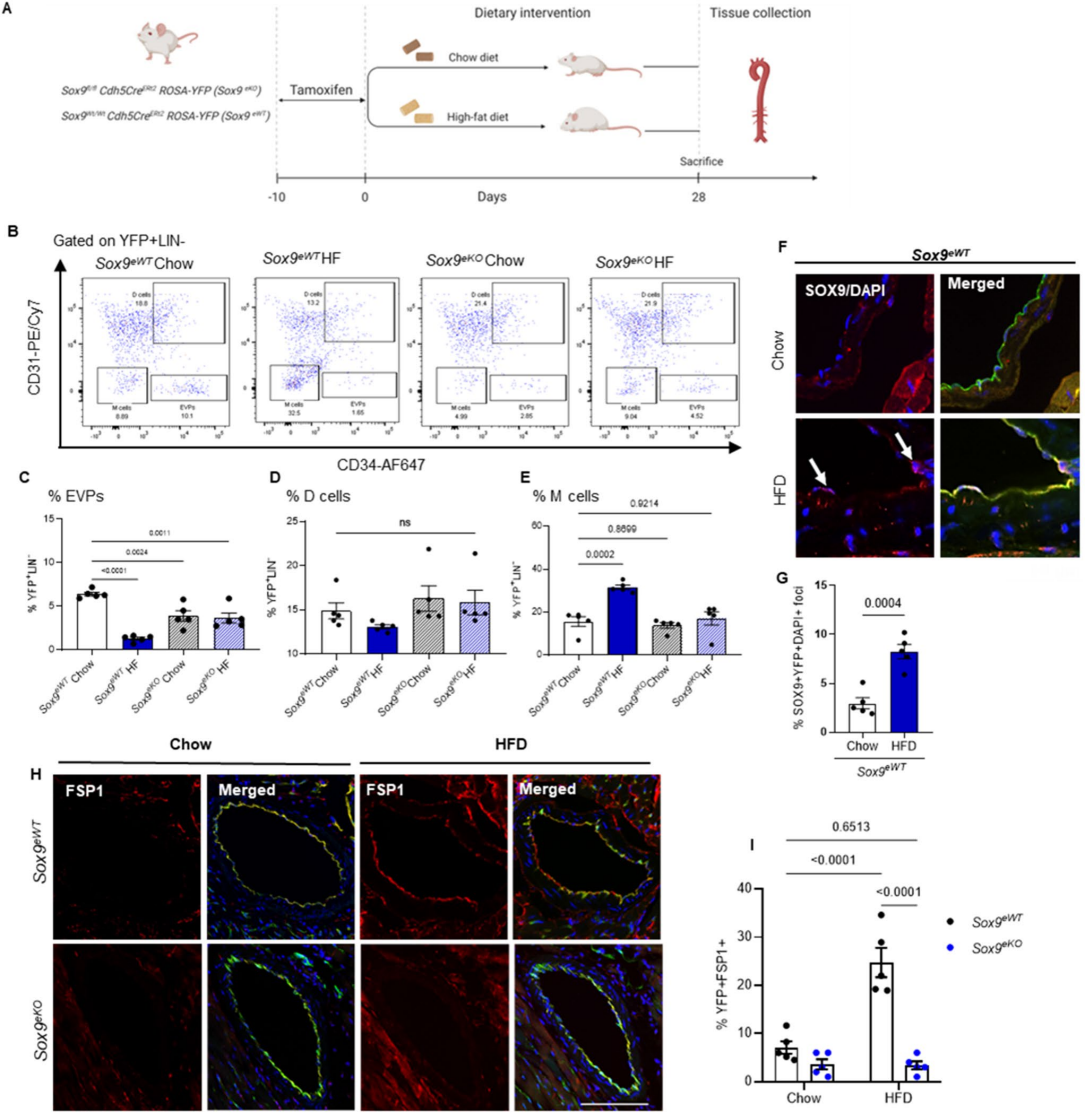
Endothelial-specific ablation of Sox9 attenuates high-fat diet-induced EndMT in vivo. (**A**) Schematic timeline and mouse model used throughout this experiment. Cdh5CreERt2 (Sox9eWT) and Sox9fl/fl/Cdh5CreErt2/ROSAYfp (Sox9eKO) were fed either the control chow or HFD. Aortic endothelial populations were then assessed 4 weeks after. (**B**) Gating strategy used to identify endothelial populations. (**C**-**E**) FACS analysis of Sox9eWT and Sox9eKO aortic endothelial populations after 4 weeks HFD. (**F**-**G**) Immunofluorescent image and quantification of endothelial SOX9 expression of Sox9eWT and Sox9eKO coronary arterial vessel after HFD. (**H**-**I**) Coronary arterial vessel co-expression of FSP-1, CD31 and Cre-YFP examined under immunofluorescence staining in Sox9eWT and Sox9eKO fed HFD. (*n* = 5; 5 biologically independent samples; mean ± SD; p value was calculated by one-way ANOVA with multiple comparison)

To assess the contribution of high fat diet (HFD) on endothelial SOX9 expression, and subsequent EndMT, mice were subjected to a 4-week HFD regime (Fig. 5A). At the end of the diet, significant increases in body weight and visceral fat mass within the HFD group was observed, regardless of genotype (*p* < 0.001 vs. Chow, E Supplemental Fig. 6A and B). Immunofluorescent examination of the coronary arterial vessels demonstrated a significant increase in nuclear expression of SOX9 in endothelial cells (CD31 + cells) of *Sox9*^*eWT*^ mice fed a HFD compared to a normal chow diet, supportive of HFD inducing the expression of SOX9 (*p* = 0.0004 vs. *Sox9*^*eWT*^ Chow, Fig. 5F and G). Next, we examined murine endovascular progenitors (EVPs) as a surrogate model of human ECFCs to evaluate progenitor activity [2,10,20–22].

The aortic endothelial hierarchy was examined under flow cytometry as previously reported by us (as controlled by fluorescence-minus-one staining, Supplemental Fig. 6C). Briefly the YFP + Lineage^neg^CD34^+^CD31^low^ fraction identified endovascular progenitors (EVP) known to carry self-renewal activity. No significant differences in *Cdh5Cre* driven-YFP + cells (representing total endothelial cells) were detected between the *Sox9*^*eWT*^ and *Sox9*^*eKO*^ groups, regardless of diet. After gating on total YFP^+^Lineage^neg^ endothelial cells, CD31 and CD34 were used to delineate the murine endothelial heterogeneity and hierarchy as previously reported [7,10,20]. In *Sox9*^*eWT*^ mice fed HFD, a significant accumulation of mesenchymal M cells (YFP + Lineage^neg^CD31-CD34-) indicative of EndMT (*p* < 0.002 vs. *Sox9*^*eWT*^ Chow, Fig. 5B and E) was observed and was associated with a depletion of EVP progenitors (*p* < 0.001 vs. *Sox9*^*eWT*^ Chow, Fig. 5B and C) corroborating the transition to a mesenchymal phenotype and loss of progenitor numbers observed in vivo. Upon *Sox9* deletion, the transition of EVPs to M cells was effectively prevented in *Sox9*^*eKO*^ group, as no significant changes were observed between the diet groups (Fig. 5B and E). However, EVP progenitor levels did not differ according to diet. Importantly, even in the absence of a high-fat diet, conditional loss of *Sox9* in the endothelium resulted in a significant reduction in the progenitor population (*p* < 0.0024 *Sox9*^*eKO*^ vs. *Sox9*^*eWT*^ Chow), recapitulating the importance of the *Sox9* gene in progenitor function and self-renewal.

Next, we used immunostaining to characterise cells labelled with YFP, witnessing their expression of the endothelial marker *cdh5* at the time of tamoxifen injection. We used a well-characterised mesenchymal factor, fibroblast-specific protein 1 (FSP-1), in addition to a key endothelial marker CD31 to examine EndMT within the coronary vasculature. The number of YFP + cells which co-expressed CD31 and FSP-1 was significantly increased in *Sox9*^*eWT*^ mice given HFD compared to chow supporting the advent of EndMT upon HFD (*p* < 0.001 vs. *Sox9*^*eWT*^ Chow, Fig. 5H and I). Equally, in support of the previous flow cytometry observations, the conditional deletion of *Sox9* within the endothelium largely attenuated HFD-induced EndMT, reflected by the reduction of endothelial FSP-1 expression.

Overall, these findings support our in vitro observation that HFD induces a mesenchymal transition associated with the loss of progenitors and the former can be inhibited effectively by the loss of *Sox9*.

## Discussion

Endothelial to mesenchymal transition is a process encountered in an array of physiological and pathological situations. It relies on the ability of endothelial cells to differentiate or transition to a mesenchymal fate [10,15,17,23–27]. This process is involved in adult pathological situation such as fibrosis and wound healing but also atherosclerosis. In this work, to improve our understanding of molecular events supporting EndMT, we used a well-established model of EndMT and report that exposure to oxLDL induced a dramatic loss in self-renewal capacity and endothelial function while it promoted mesenchymal attributes of increased motility and contractility. Systematic evaluation of chromatin accessibility uncovered substantial changes occurring during the EndMT process driving the gene expression alterations. Importantly, during EndMT, chromatin accessibility of endothelial genes persisted while access to mesenchymal genes increased. This supported our observation that the EndMT process was reversible in most cells in endothelial culture conditions. SOX9 was identified as a key transcription factor with over-represented binding motifs in genomic areas with changed accessibility upon EndMT. Indeed, loss of SOX9 in vivo and in vitro largely prevented EndMT associated mesenchymal gene expression and functional changes related to oxidized LDL or HFD. These findings define SOX9 as a key driver of EndMT associated mesenchymal traits.

As previously reported, oxLDL treatment of ECFCs triggered a range of observations consistent with EndMT such as losses in endothelial CD34 [28,29], gain in expression of CD90[15,30] as well as a range of functional changes such as increased motility, contractility, and reduced tube-like formation capacity. Morphologically, endothelial cells undergoing EndMT adopted a spindle shape. These findings were further supported in vivo in animals exposed to a high-fat diet demonstrating mesenchymal transition of endothelial cells through lineage tracing. Overall, our findings contribute in vivo and in vitro to the body of work suggesting the advent of EndMT in endothelial cells exposed to oxLDL. Our study however brought novel insights into the mechanisms driving EndMT: the loss of self-renewal capacity, chromatin accessibility changes and finally the importance of SOX9 in this process were key additions to our expanding understanding of EndMT.

The significant reduction in ECFC self-renewal capacity as demonstrated by the reduction in high proliferative potential colonies was further supported by the loss of EVPs in vivo in animals having received a high fat diet. In both conditions a significant portion of the cells exposed to oxLDL or HFD remained endothelial. Only a small percentage of ECFCs treated with oxLDL expressed CD90 or lost both CD31 and CD34 while maintaining the lineage marker YFP. This data is suggestive that oxLDL may differentially influence EndMT within a population of ECFCs, with subpopulations exhibiting increased susceptibility [31]. We and others have reported that ECFC self-renewal relies on a reduced number of cells that have the capacity to form HPP colonies [32]. Given the restricted expression of *Sox9* to EVPs in vivo, it could be argued that EndMT occurs favourably in cells that have progenitor function [10]. This hypothesis is also compatible with the observation of human bipotent progenitors in vivo able to give rise to endothelial and mesenchymal progenies [19]. However, there are significant differences in the behaviour of endothelial cells between mice and human and the relevance of our observations in vivo in humans remains to be demonstrated. Overall, the parallel reduction in self-renewal and transition to a mesenchymal phenotype could be due to the terminal differentiation of progenitors towards a mesenchymal fate. A key issue in high-fat diet or oxLDL induced EndMT could emanate from progenitor depletion and loss of self-renewal capacity associated with preferential mesenchymal differentiation and fate choice which on the long term may result in pathologies. Whether attenuation of EndMT through inhibition of SOX9 can impact the development of atherosclerotic plaques remains to be explored as our experimental set-up only exposed mice to a short duration of high-fat diet.

Global assessment through bulk RNA and ATAC sequencing revealed significant shifts in both the transcriptomic and chromatin landscape, supporting the activation of EndMT associated pathways. Importantly, these studies showed that EndMT upon oxLDL exposure reduced the expression of endothelial genes without reducing their chromatin accessibility suggesting some reversibility. Specifically, *SOX9* was amongst the top genes upregulated in ECFCs post oxLDL exposure. This was further supported by in vivo increased expression of SOX9. Further support of EndMT and aberrant *SOX9* expression was the loss of Notch1 signalling pathway constituents *DLL4, HEY1* and *SOX17* [9,10,33]. As demonstrated previously, Notch signalling is crucial in maintaining homeostatic regulation of *SOX9* [34], and preventing endothelial progenitors entering EndMT [10]. Consistently, endothelial *Notch1* has also been demonstrated to be suppressed by inflammatory lipids, resulting in increased monocyte adhesion and more severe HFD induced atherosclerosis [35]. Beyond SOX9, well characterised EndMT associated pathways, such as the TGFβ and TNFα signalling pathways were up-regulated in oxLDL treated ECFCs [16,36–39]. Consistent with previous studies, oxLDL treatment of HAECs induced the expression of TGFβ receptor-1 mediated through the oxLDL-receptor 1 (OLR1) pathway. OLR1/LOX1, a scavenger receptor for oxLDL metabolism has been shown to contribute to cell migration and transformation in response to stimulating inflammatory signals as captured in both RNAseq (TNFa pathway activation) and ATACseq (NFkB motifs). Additionally, OLR1 activation is also associated with increased collagen production in myofibroblasts [40–42]. Supportive of this, functional assessment of oxLDL treated ECFCs demonstrated increased cell contractility and migratory functions. Further, increased TGFβ signalling has also been shown to phosphorylate and stabilise SOX9 in a SMAD dependent manner [43]. Overall, it could be argued that *SOX9* overexpression is not a primary event and is the result of multiple pathways directly activated by oxLDL. However, SOX9 might integrate the signals from these pathways and play an important effector role.

The precise role of oxLDL induced SOX9 activation and ECFC EndMT was explored through an inducible shRNA knockdown model. The inducible knockdown of *SOX9* led to the attenuation of all examined EndMT associated gene expression changes. This is suggestive that the upregulation of SOX9 post oxLDL exposure is key in driving the expression or direct translation of key mesenchymal factors, namely *SNAI1* and *RUNX2*, which have been reported to be regulated by SOX9[44,45], and is also central to EndMT under atherogenic conditions [13,46]. Alternatively, the knockdown of *SOX9* also resulted in the increase of endothelial junctional gene *CDH5* as well as *Notch1* ligand *DLL4*. This is suggestive that endothelial phenotype, at the molecular level is maintained through either the attenuation of mesenchymal factor expression, or the restoration of Notch signalling, which was previously demonstrated to be crucial in facilitating endothelial repair [9,10,47]. These findings recapitulate previous observations in cutaneous wound models where *Sox9* and Notch signalling played opposing roles in driving and protecting against EndMT induced wound fibrosis [10] Overall, the importance of *SOX9* in EndMT is again demonstrated in the setting of exposure to oxLDL.

Although *SOX9* silencing demonstrated robust restoration of the endothelial gene expression in ECFCs, it was found that endothelial progenitor-related functions, such as capillary formation [48] and self-renewal capacities [4,5], inhibited through oxLDL treatment, were not improved. This may be related to the importance of *SOX9* in the endothelium during homeostasis. Indeed, *Sox9* conditional knock-out has been reported to reduce progenitor numbers in vivo [10] as it was also shown here in vitro and *in vivo.* Accordingly, this finding demonstrates that the attenuation of EndMT, at both the molecular and functional levels cannot be directly associated with restoring endothelial progenitor function if SOX9 is inhibited. On the other hand, although *SOX9* is expressed at low levels in homeostatic ECFCs, its baseline expression may be needed to maintain endothelial progenitor stemness as observed in other systems [49–51]. Interestingly, similarly to results observed here, low homeostatic levels of SOX9 expression in intestinal epithelial stem cells supports their proliferative self-renewal capacity, while SOX9 overexpression suppressed proliferation and induced morphological changes [50]. In accordance, SOX9 binding motifs were associated with some of the downregulated chromosomal accessibility peaks. It is possible that these peaks are not restored upon *Sox9* knock-out resulting in a lasting effect of *SOX9* overexpression. Therefore, the results presented here highlight the idea that inhibition of SOX9 to reduce EndMT does not necessarily restore endothelial progenitor function and needs to be taken into consideration during the development of future targeted therapies.

The identification of the molecular network driving EndMT presents an exciting new avenue in understanding this important phenomenon in physiology and pathology. The results presented here identified SOX9 up-regulation as a key step during EndMT, with its specific silencing significantly attenuating the mesenchymal gene expression of functions resulting from this process. These findings may open new avenues for targeting EndMT through its molecular mechanism.

### Electronic supplementary material

Below is the link to the electronic supplementary material.

Supplementary Material 1

## Data Availability

RNA-Seq and ATAC-Seq data were deposited into the Gene Expression Omnibus database under accession number GSE247712; subseries GSE247709 (ATAC-Seq) and GSE247711 (RNA-Seq); and are available at the following URL: https://www.ncbi.nlm.nih.gov/geo/query/acc.cgi?acc=GSE247709, https://www.ncbi.nlm.nih.gov/geo/query/acc.cgi?acc=GSE247711.

## References

[1] Banno K, Yoder MC (2018) Tissue regeneration using endothelial colony-forming cells: promising cells for vascular repair. Pediatr Res 83:283–290 28915234 10.1038/pr.2017.231

[2] Dight J, Zhao J, Styke C, Khosrotehrani K, Patel J (2021) Resident vascular endothelial progenitor definition and function: the age of reckoning. Angiogenesis 10.1007/s10456-021-09817-2 PMC8813834 34499264

[3] Patel J, Seppanen E, Chong MS, Yeo JS, Teo EY, Chan JK, Fisk NM, Khosrotehrani K (2013) Prospective surface marker-based isolation and expansion of fetal endothelial colony-forming cells from human term placenta. Stem Cells Transl Med 2:839–847 24106336 10.5966/sctm.2013-0092 PMC3808199

[4] Ingram DA, Mead LE, Moore DB, Woodard W, Fenoglio A, Yoder MC (2005) Vessel wall-derived endothelial cells rapidly proliferate because they contain a complete hierarchy of endothelial progenitor cells. Blood 105:2783–2786 15585655 10.1182/blood-2004-08-3057

[5] Yoder MC, Mead LE, Prater D, Krier TR, Mroueh KN, Li F, Krasich R, Temm CJ, Prchal JT, Ingram DA (2007) Redefining endothelial progenitor cells via clonal analysis and hematopoietic stem/progenitor cell principals. Blood 109:1801–1809 17053059 10.1182/blood-2006-08-043471 PMC1801067

[6] Lin Y, Banno K, Gil CH, Myslinski J, Hato T, Shelley WC, Gao H, Xuei X, Liu Y, Basile DP, Yoshimoto M, Prasain N, Tarnawsky SP, Adams RH, Naruse K, Yoshida J, Murphy MP (2023) Horie K and Yoder MC. Origin, prospective identification, and function of circulating endothelial colony-forming cells in mice and humans. JCI Insight. ;8 10.1172/jci.insight.164781 PMC10077473 36692963

[7] Patel J, Seppanen EJ, Rodero MP, Wong HY, Donovan P, Neufeld Z, Fisk NM, Francois M, Khosrotehrani K (2017) Functional definition of progenitors Versus mature endothelial cells reveals key SoxF-Dependent differentiation process. Circulation 135:786–805 27899395 10.1161/CIRCULATIONAHA.116.024754

[8] Xu Y, Kovacic JC (2023) Endothelial to Mesenchymal Transition in Health and Disease. Annu Rev Physiol 85:245–267 36266259 10.1146/annurev-physiol-032222-080806

[9] Patel J, Baz B, Wong HY, Lee JS, Khosrotehrani K (2018) Accelerated endothelial to mesenchymal transition increased fibrosis via deleting Notch Signaling in Wound vasculature. J Invest Dermatol 138:1166–1175 29248546 10.1016/j.jid.2017.12.004

[10] Zhao J, Patel J, Kaur S, Sim S-L, Wong HY, Styke C, Hogan I, Kahler S, Hamilton H, Wadlow R, Dight J, Hashemi G, Sormani L, Roy E, Yoder MC, Francois M, Khosrotehrani K (2021) Sox9 and Rbpj differentially regulate endothelial to mesenchymal transition and wound scarring in murine endovascular progenitors. Nat Commun 12:2564 33963183 10.1038/s41467-021-22717-9 PMC8105340

[11] Nano R, Sim SL, Shafiee A, Khosrotehrani K, Patel J (2022) High-yield isolation of pure fetal endothelial colony forming cells and mesenchymal stem cells from the human full-term placenta. STAR Protoc 3:101354 35509970 10.1016/j.xpro.2022.101354 PMC9059155

[12] Sallets A, Robinson S, Kardosh A, Levy R (2018) Enhancing immunotherapy of STING agonist for lymphoma in preclinical models. Blood Adv 2:2230–2241 30194137 10.1182/bloodadvances.2018020040 PMC6134215

[13] Su Q, Sun Y, Ye Z, Yang H, Li L (2018) Oxidized low density lipoprotein induces endothelial-to-mesenchymal transition by stabilizing snail in human aortic endothelial cells. Biomed Pharmacother 106:1720–1726 30119247 10.1016/j.biopha.2018.07.122

[14] Jia W, Wang Z, Gao C, Wu J, Wu Q (2021) Trajectory modeling of endothelial-to-mesenchymal transition reveals galectin-3 as a mediator in pulmonary fibrosis. Cell Death Dis 12:327 33771973 10.1038/s41419-021-03603-0 PMC7998015

[15] Pinto MT, Ferreira Melo FU, Malta TM, Rodrigues ES, Plaça JR, Silva WA Jr., Panepucci RA, Covas DT, de Oliveira Rodrigues C, Kashima S (2018) Endothelial cells from different anatomical origin have distinct responses during SNAIL/TGF-β2-mediated endothelial-mesenchymal transition. Am J Transl Res 10:4065–4081 30662651 PMC6325528

[16] Chen P-Y, Qin L, Li G, Wang Z, Dahlman JE, Malagon-Lopez J, Gujja S, Cilfone NA, Kauffman KJ, Sun L, Sun H, Zhang X, Aryal B, Canfran-Duque A, Liu R, Kusters P, Sehgal A, Jiao Y, Anderson DG, Gulcher J, Fernandez-Hernando C, Lutgens E, Schwartz MA, Pober JS, Chittenden TW (2019) Tellides G and Simons M. Endothelial TGF-β signalling drives vascular inflammation and atherosclerosis. Nat Metabolism 1:912–926 10.1038/s42255-019-0102-3 PMC6767930 31572976

[17] Cooley BC, Nevado J, Mellad J, Yang D, St Hilaire C, Negro A, Fang F, Chen G, San H, Walts AD, Schwartzbeck RL, Taylor B, Lanzer JD, Wragg A, Elagha A, Beltran LE, Berry C, Feil R, Virmani R, Ladich E, Kovacic JC, Boehm M (2014) TGF-β signaling mediates endothelial-to-mesenchymal transition (EndMT) during vein graft remodeling. Sci Transl Med 6:227ra34–227ra34 24622514 10.1126/scitranslmed.3006927 PMC4181409

[18] Chen H, Li D, Saldeen T, Mehta JL (2001) Transforming growth factor-beta(1) modulates oxidatively modified LDL-induced expression of adhesion molecules: role of LOX-1. Circ Res 89:1155–1160 11739280 10.1161/hh2401.100598

[19] Shafiee A, Patel J, Hutmacher DW, Fisk NM, Khosrotehrani K (2018) Meso-Endothelial Bipotent progenitors from Human Placenta Display distinct Molecular and Cellular Identity. Stem Cell Rep 10:890–904 10.1016/j.stemcr.2018.01.011 PMC5918195 29478891

[20] Donovan P, Patel J, Dight J, Wong HY, Sim SL, Murigneux V, Francois M, Khosrotehrani K (2019) Endovascular progenitors infiltrate melanomas and differentiate towards a variety of vascular beds promoting tumor metastasis. Nat Commun 10:18 30604758 10.1038/s41467-018-07961-w PMC6318267

[21] Lukowski SW, Patel J, Andersen SB, Sim SL, Wong HY, Tay J, Winkler I, Powell JE, Khosrotehrani K (2019) Single-cell transcriptional profiling of aortic endothelium identifies a Hierarchy from Endovascular progenitors to differentiated cells. Cell Rep 27:2748–2758e3 31141696 10.1016/j.celrep.2019.04.102

[22] Patel J, Donovan P, Khosrotehrani K (2016) Concise Review: functional definition of endothelial progenitor cells: a molecular perspective. Stem Cells Transl Med 5:1302–1306 27381992 10.5966/sctm.2016-0066 PMC5031185

[23] Evrard SM, Lecce L, Michelis KC, Nomura-Kitabayashi A, Pandey G, Purushothaman KR, d’Escamard V, Li JR, Hadri L, Fujitani K, Moreno PR, Benard L, Rimmele P, Cohain A, Mecham B, Randolph GJ, Nabel EG, Hajjar R, Fuster V, Boehm M, Kovacic JC (2016) Endothelial to mesenchymal transition is common in atherosclerotic lesions and is associated with plaque instability. Nat Commun 7:11853 27340017 10.1038/ncomms11853 PMC4931033

[24] Kovacic JC, Dimmeler S, Harvey RP, Finkel T, Aikawa E, Krenning G, Baker AH (2019) Endothelial to mesenchymal transition in Cardiovascular Disease: JACC State-of-the-art review. J Am Coll Cardiol 73:190–209 30654892 10.1016/j.jacc.2018.09.089 PMC6865825

[25] Moonen J-RAJ, Krenning G, Brinker MGL, Koerts JA, van Luyn MJA, Harmsen MC (2010) Endothelial progenitor cells give rise to pro-angiogenic smooth muscle-like progeny. Cardiovascular Res 86:506–515 10.1093/cvr/cvq012 20083576

[26] Wang Z, Han Z, Tao J, Wang J, Liu X, Zhou W, Xu Z, Zhao C, Wang Z, Tan R, Gu M (2017) Role of endothelial-to-mesenchymal transition induced by TGF-β1 in transplant kidney interstitial fibrosis. J Cell Mol Med 21:2359–2369 28374926 10.1111/jcmm.13157 PMC5618680

[27] Zeisberg EM, Tarnavski O, Zeisberg M, Dorfman AL, McMullen JR, Gustafsson E, Chandraker A, Yuan X, Pu WT, Roberts AB, Neilson EG, Sayegh MH, Izumo S, Kalluri R (2007) Endothelial-to-mesenchymal transition contributes to cardiac fibrosis. Nat Med 13:952–961 17660828 10.1038/nm1613

[28] Choi S-H, Kim AR, Nam J-K, Kim J-M, Kim J-Y, Seo HR, Lee H-J, Cho J, Lee Y-J (2018) Tumour-vasculature development via endothelial-to-mesenchymal transition after radiotherapy controls CD44v6 + cancer cell and macrophage polarization. Nat Commun 9:5108 30504836 10.1038/s41467-018-07470-w PMC6269447

[29] Suzuki T, Carrier EJ, Talati MH, Rathinasabapathy A, Chen X, Nishimura R, Tada Y, Tatsumi K, West J (2017) Isolation and characterization of endothelial-to-mesenchymal transition cells in pulmonary arterial hypertension. Am J Physiology-Lung Cell Mol Physiol 314:L118–L126 10.1152/ajplung.00296.2017 PMC5866427 28935639

[30] Medici D, Kalluri R (2012) Endothelial-mesenchymal transition and its contribution to the emergence of stem cell phenotype. Semin Cancer Biol 22:379–384 22554794 10.1016/j.semcancer.2012.04.004 PMC3422405

[31] Farrar EJ, Butcher JT (2014) Heterogeneous susceptibility of valve endothelial cells to mesenchymal transformation in response to TNFα. Ann Biomed Eng 42:149–161 23982279 10.1007/s10439-013-0894-3 PMC3905205

[32] Patel J, Wong HY, Wang W, Alexis J, Shafiee A, Stevenson AJ, Gabrielli B, Fisk NM, Khosrotehrani K (2016) Self-Renewal and high proliferative colony forming capacity of late-outgrowth endothelial progenitors is regulated by cyclin-dependent kinase inhibitors driven by Notch Signaling. Stem Cells 34:902–912 26732848 10.1002/stem.2262

[33] Chen S, Tao J, Bae Y, Jiang MM, Bertin T, Chen Y, Yang T, Lee B (2013) Notch gain of function inhibits chondrocyte differentiation via Rbpj-dependent suppression of Sox9. J Bone Min Res 28:649–659 10.1002/jbmr.1770 PMC3548081 22991339

[34] Briot A, Jaroszewicz A, Warren CM, Lu J, Touma M, Rudat C, Hofmann JJ, Airik R, Weinmaster G, Lyons K, Wang Y, Kispert A, Pellegrini M, Iruela-Arispe ML (2014) Repression of Sox9 by Jag1 is continuously required to suppress the default chondrogenic fate of vascular smooth muscle cells. Dev Cell 31:707–721 25535917 10.1016/j.devcel.2014.11.023 PMC4311887

[35] Briot A, Civelek M, Seki A, Hoi K, Mack JJ, Lee SD, Kim J, Hong C, Yu J, Fishbein GA, Vakili L, Fogelman AM, Fishbein MC, Lusis AJ, Tontonoz P, Navab M, Berliner JA, Iruela-Arispe ML (2015) Endothelial NOTCH1 is suppressed by circulating lipids and antagonizes inflammation during atherosclerosis. J Exp Med 212:2147–2163 26552708 10.1084/jem.20150603 PMC4647265

[36] Souilhol C, Serbanovic-Canic J, Fragiadaki M, Chico TJ, Ridger V, Roddie H, Evans PC (2020) Endothelial responses to shear stress in atherosclerosis: a novel role for developmental genes. Nat Reviews Cardiol 17:52–63 10.1038/s41569-019-0239-5 31366922

[37] Ma J, Sanchez-Duffhues G, Goumans M-J, ten Dijke P (2020) TGF-β-Induced endothelial to mesenchymal transition in Disease and tissue Engineering. Front Cell Dev Biology. ;8 10.3389/fcell.2020.00260 PMC7187792 32373613

[38] Yoshimatsu Y, Kimuro S, Pauty J, Takagaki K, Nomiyama S, Inagawa A, Maeda K, Podyma-Inoue KA, Kajiya K, Matsunaga YT, Watabe T (2020) TGF-beta and TNF-alpha cooperatively induce mesenchymal transition of lymphatic endothelial cells via activation of activin signals. PLoS ONE 15:e0232356 32357159 10.1371/journal.pone.0232356 PMC7194440

[39] Adjuto-Saccone M, Soubeyran P, Garcia J, Audebert S, Camoin L, Rubis M, Roques J, Binétruy B, Iovanna JL, Tournaire R (2021) TNF-α induces endothelial–mesenchymal transition promoting stromal development of pancreatic adenocarcinoma. Cell Death Dis 12:649 34172716 10.1038/s41419-021-03920-4 PMC8233393

[40] Hu C, Dandapat A, Sun L, Khan JA, Liu Y, Hermonat PL, Mehta JL (2008) Regulation of TGFbeta1-mediated collagen formation by LOX-1: studies based on forced overexpression of TGFbeta1 in wild-type and lox-1 knock-out mouse cardiac fibroblasts. J Biol Chem 283:10226–10231 18182394 10.1074/jbc.M708820200

[41] Jiang L, Jiang S, Zhou W, Huang J, Lin Y, Long H, Luo Q (2019) Oxidized low density lipoprotein receptor 1 promotes lung metastases of osteosarcomas through regulating the epithelial-mesenchymal transition. J Transl Med 17:369–369 31718700 10.1186/s12967-019-2107-9 PMC6852786

[42] Villa M, Cerda-Opazo P, Jimenez-Gallegos D, Garrido-Moreno V, Chiong M, Quest AFG, Toledo J, Garcia L (2020) Pro-fibrotic effect of oxidized LDL in cardiac myofibroblasts. Biochem Biophys Res Commun 524:696–701 32033750 10.1016/j.bbrc.2020.01.156

[43] Coricor G, Serra R (2016) TGF-β regulates phosphorylation and stabilization of Sox9 protein in chondrocytes through p38 and smad dependent mechanisms. Sci Rep 6:38616 27929080 10.1038/srep38616 PMC5144132

[44] Choi B-J, Park S-A, Lee S-Y, Cha YN, Surh Y-J (2017) Hypoxia induces epithelial-mesenchymal transition in colorectal cancer cells through ubiquitin-specific protease 47-mediated stabilization of snail: a potential role of Sox9. Sci Rep 7:15918 29162839 10.1038/s41598-017-15139-5 PMC5698333

[45] Cheng A, Genever PG (2010) SOX9 determines RUNX2 transactivity by directing intracellular degradation. J Bone Miner Res 25:2680–2689 20593410 10.1002/jbmr.174

[46] Souilhol C, Harmsen MC, Evans PC, Krenning G (2018) Endothelial–mesenchymal transition in atherosclerosis. Cardiovascular Res 114:565–577 10.1093/cvr/cvx253 29309526

[47] McDonald AI, Shirali AS, Aragón R, Ma F, Hernandez G, Vaughn DA, Mack JJ, Lim TY, Sunshine H, Zhao P, Kalinichenko V, Hai T, Pelegrini M, Ardehali R, Iruela-Arispe ML (2018) Endothelial regeneration of large vessels is a biphasic process driven by local cells with distinct proliferative capacities. Cell Stem Cell 23:210–225e6 30075129 10.1016/j.stem.2018.07.011 PMC6178982

[48] Peters EB (2018) Endothelial progenitor cells for the vascularization of Engineered tissues. Tissue Eng Part B Rev 24:1–24 28548628 10.1089/ten.teb.2017.0127 PMC5797330

[49] Akiyama H, Chaboissier MC, Martin JF, Schedl A, de Crombrugghe B (2002) The transcription factor Sox9 has essential roles in successive steps of the chondrocyte differentiation pathway and is required for expression of Sox5 and Sox6. Genes Dev 16:2813–2828 12414734 10.1101/gad.1017802 PMC187468

[50] Formeister EJ, Sionas AL, Lorance DK, Barkley CL, Lee GH, Magness ST (2009) Distinct SOX9 levels differentially mark stem/progenitor populations and enteroendocrine cells of the small intestine epithelium. Am J Physiol Gastrointest Liver Physiol 296:G1108–G1118 19228882 10.1152/ajpgi.00004.2009 PMC2696217

[51] Pritchett J, Athwal V, Roberts N, Hanley NA, Hanley KP (2011) Understanding the role of SOX9 in acquired diseases: lessons from development. Trends Mol Med 17:166–174 21237710 10.1016/j.molmed.2010.12.001

