# Supplementary material for: Distinct roles of SOX9 in self-renewal of progenitors and mesenchymal transition of the endothelium
Source: Angiogenesis. 2024 May 11;27(3):545–60. doi: 10.1007/s10456-024-09927-7 (PMC11303482; doi:10.1007/s10456-024-09927-7)
Supplement: Supplementary file 1 — Supplementary Material 1 [file 10456_2024_9927_MOESM1_ESM.docx]

**Supplemental Fig. 1. Reversibility of CD90 Surface Marker Expression. (A) Experimental timeline and design schematic. ECFCs were treated with either vehicle or oxLDL (25 µg/mL) for five days. CD90-positive oxLDL-treated ECFCs were FACS sorted, cultured for 14 days in EGM2 medium without oxLDL, and subsequently evaluated for CD90 expression using flow cytometry. (B) Flow cytometry analysis results (n = 3 ECFCs isolated from biologically independent donors; mean ± SD; p-value calculated using one-way ANOVA with multiple comparisons).**

**Supplemental Fig. 2. RNAseq dataset comparison and GSEA.** (A and B) Gene Set Enrichment Analysis (GSEA) plot for oxLDL-enriched hallmark Hypoxia and TNFalpha signalling (Molecular Signatures Database, MSigDB) showing the profile of the running Enrichment Score (ES) and positions of gene set members on the rank ordered list. NES (top), and FDR q value (bottom rows) are indicated on GSEA plot. (C and D) Cross comparison between oxLDL vs. Veh and ECFC precursors vs. MSC precursors datasets from previously published bulk RNA sequencing dataset comparing human primary placental cells that gave rise in culture to ECFCs and mesenchymal stem cells (MSC). (E-H) GSEA cell signatures of genes downregulated (left) and upregulated (right) in oxLDL treated ECFCs.

**Supplemental Fig. 3. ATAC-sequencing quality controls and analysis.** (A) Heatmap showing the clusters based on the peaks, significantly different between treatment and controls (B) Principal component analysis. (C) ATAC-seq differential peaks distribution. (D) Profile of global enrichment for ATAC-seq peaks demonstrating chromatin accessibility at genome-wide transcription start sites (TSS) ± 1 kb and at up-regulated gene promoters ± 1 kb in each biological replicate per condition. (E) Pathways and (F) Cell type gene set enrichment analysis from the list of up regulated genes in oxLDL treated cells containing one or more SOX9 binding motif overlapping open chromatin peaks in their promoter region (within 5kb) (G) Transcription factor motifs analysis showing the motifs found in treatment-dependent ATAC-seq peaks (n = 3633). The ratio is the effect size, and color indicates statistical significance.

**Supplemental Fig. 4. ECFCshSOX9 generation and ECFC functional characterisation** (A) Immunoblotting of SOX9 in varying concentration of tetracycline in both ECFCshScr and ECFCshSOX9. The presence of the Tet repressor protein (20 kDa) was verified to confirm the successful lentiviral transduction. (B) Protein and mRNA (C) expression level of SOX9 quantified by Western Blot and qPCR in ECFCshSOX9 compared to ECFCshScr in varying concentration of tetracycline.

**Supplemental Fig. 5** **SOX9 overexpression in ECFCs induces progenitor dysfunction and EndMT (A) SOX9 mRNA expression assessed by qPCR. (B) COL2A1 and mesenchymal genes (FSP1, SNAI1 and RUNX2) mRNA expression assessed by qPCR. (C) Morphological differences between ECFCs overexpressing SOX9 and control ECFCs (EV). (D) Single-cell colony-forming assay. ECFCs were FACS sorted at a single-cell level and cultured for 14 days (vs EV; n = 5 experimental replicates, mean ± SD; p value was calculated by two-way ANOVA with multiple comparison to EV). (E) CD90 surface marker expression assessed by flow cytometry in 2 biologically independent ECFC donors overexpressing SOX9 vs EV. (F) Capillary tube formation assessment of EV and OvSOX9 ECFCs. (G) Capillary networks quantification using ImageJ Angiogenesis Analyzer software.**

**Supplemental Fig. 6. EVP hierarchy FMO controls and HFD-induced weight gains**. (A) Percentage weight gain of each respective mouse group demonstrated significant diet dependent increase within the HFD group compared to chow. Endothelial deletion of Sox9 did not significantly alter weight gain patterns compared to Sox9WT. (B) The gonadal fat pad weight was used to examine relative changes to visceral adipose tissue accumulation. HFD resulted in the significant increase of gonadal fat pad weight compared to control chow. (C) FMO of markers used to determine gating strategies of the murine endothelial hierarchy. Red dotted line signifies cut offs for positive populations.

**Supplemental Figures**



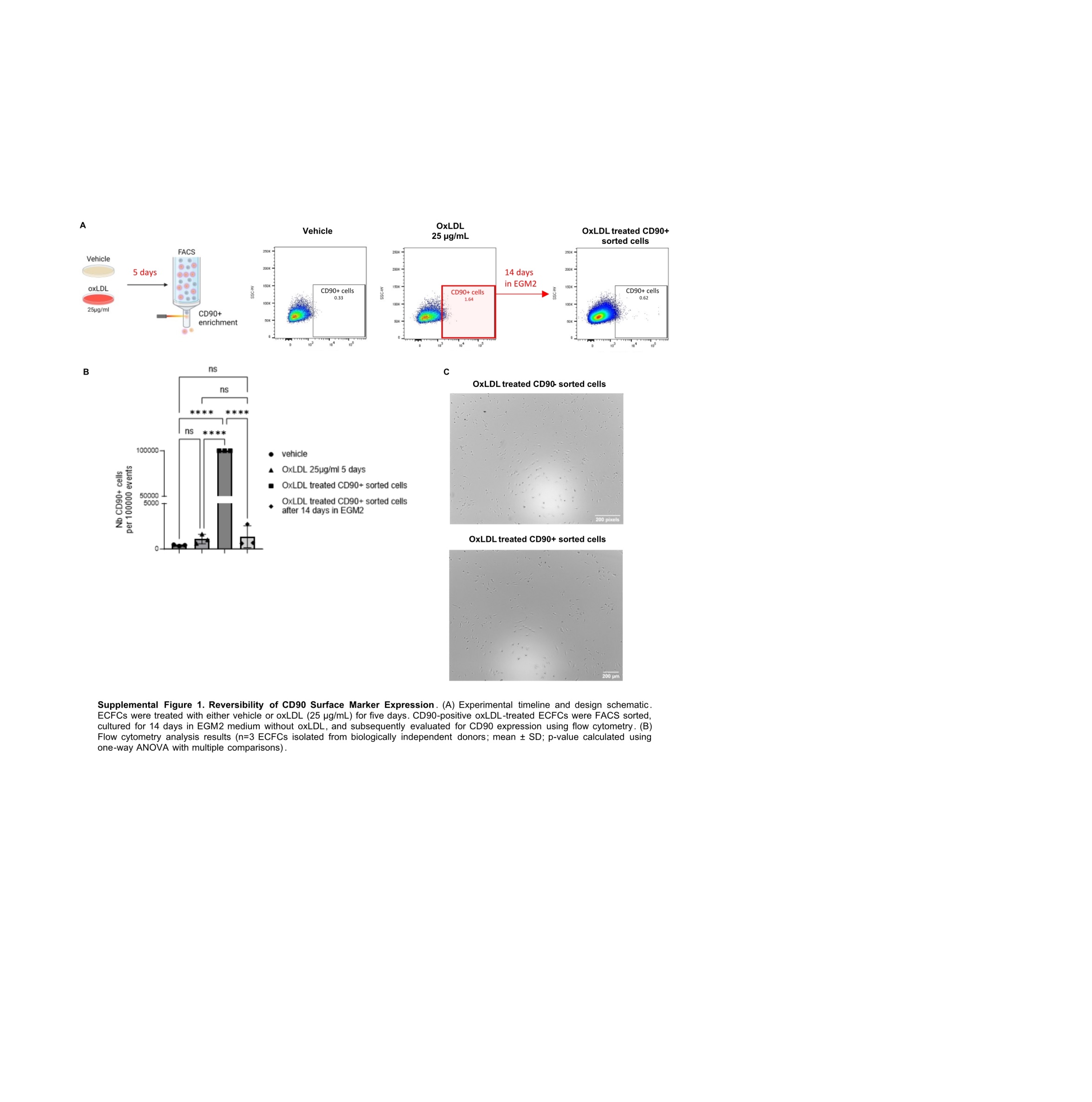



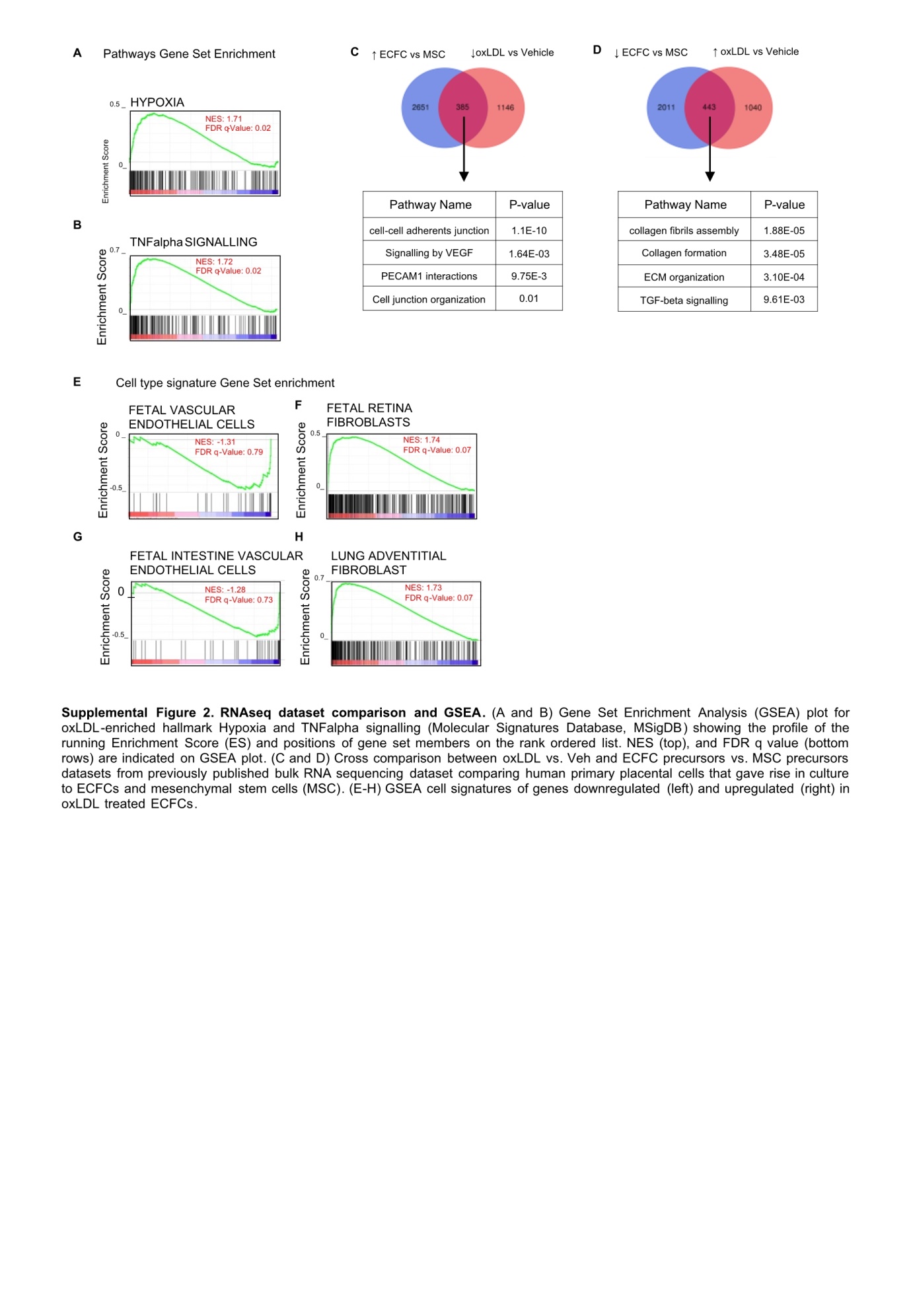



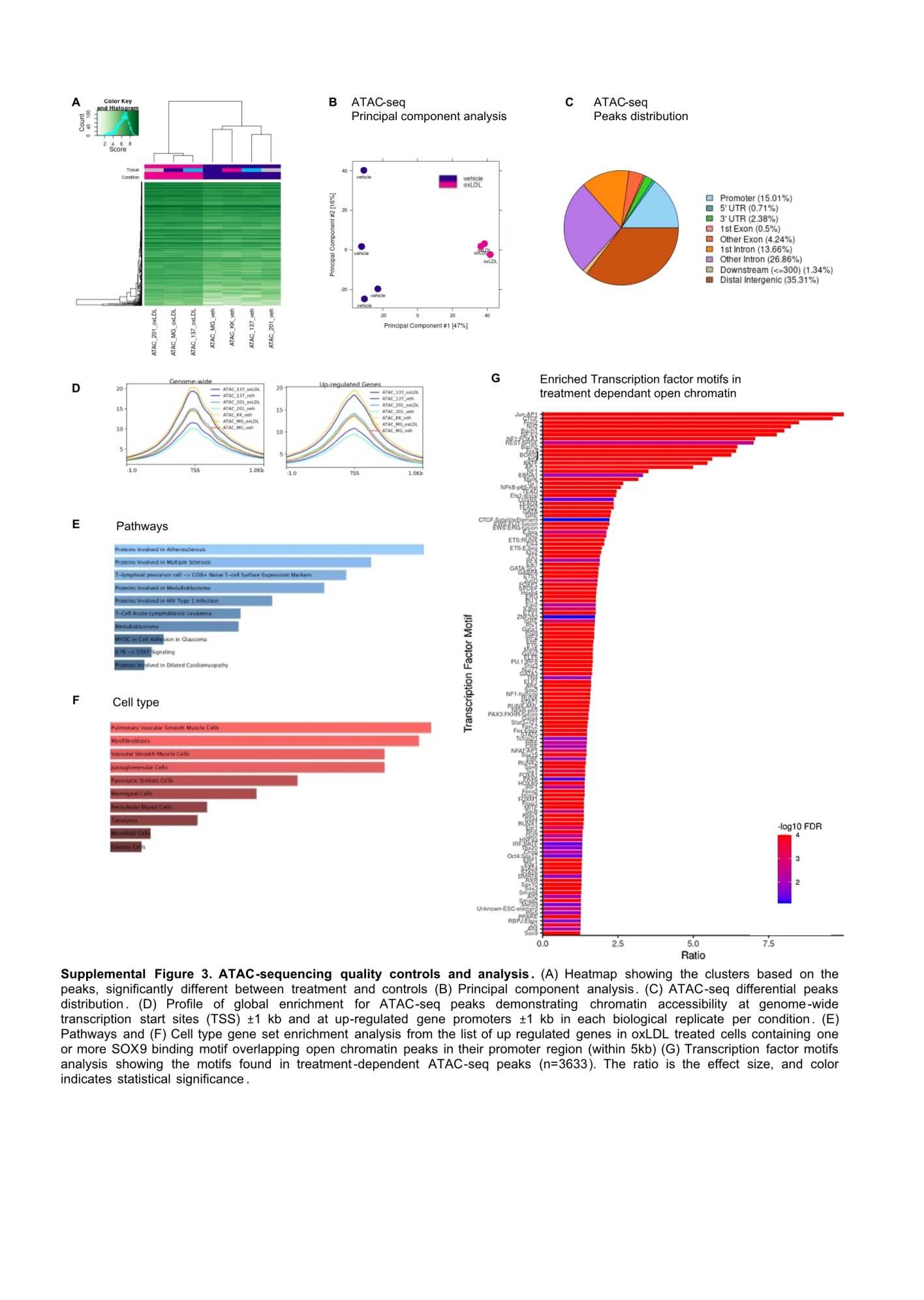



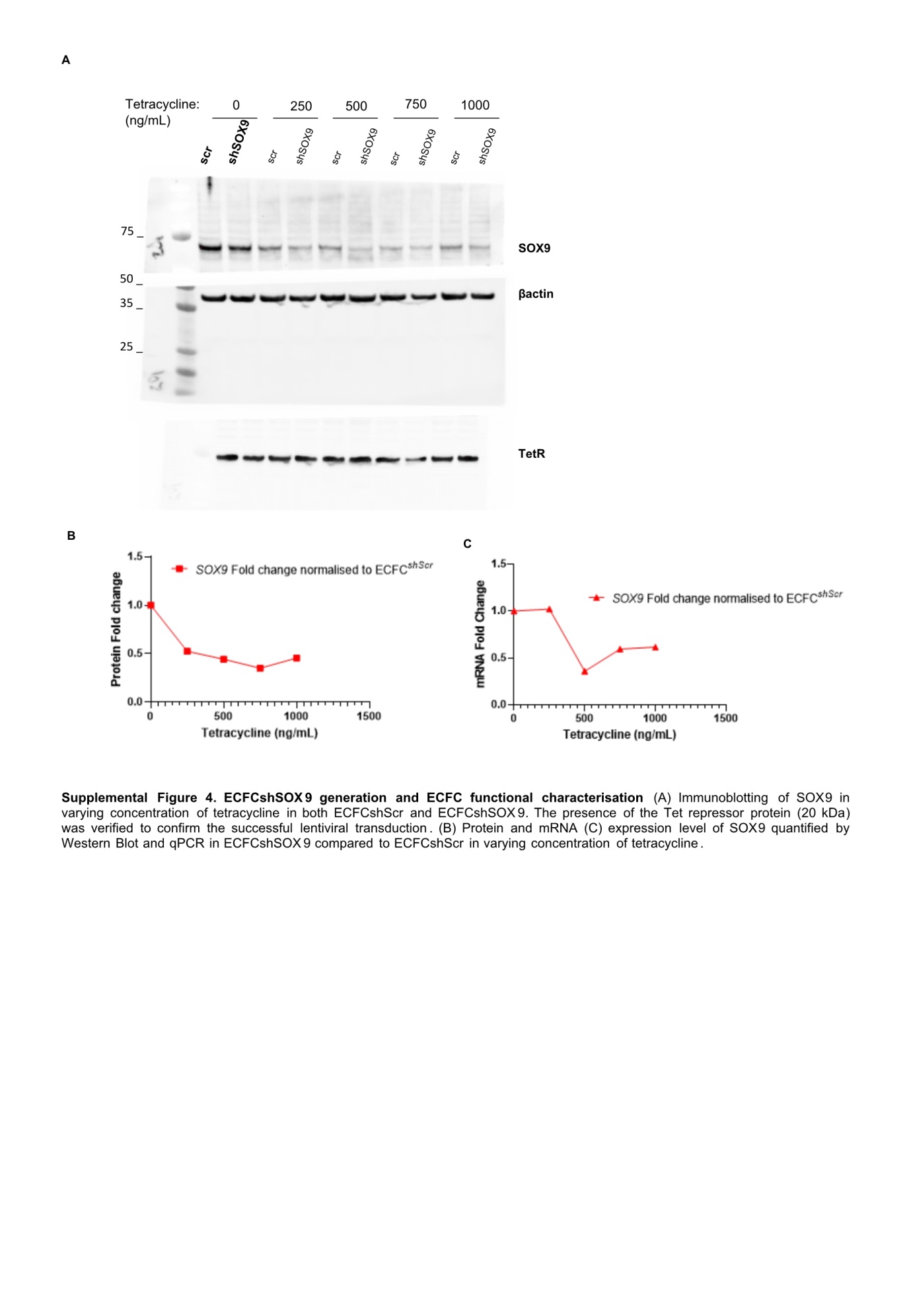


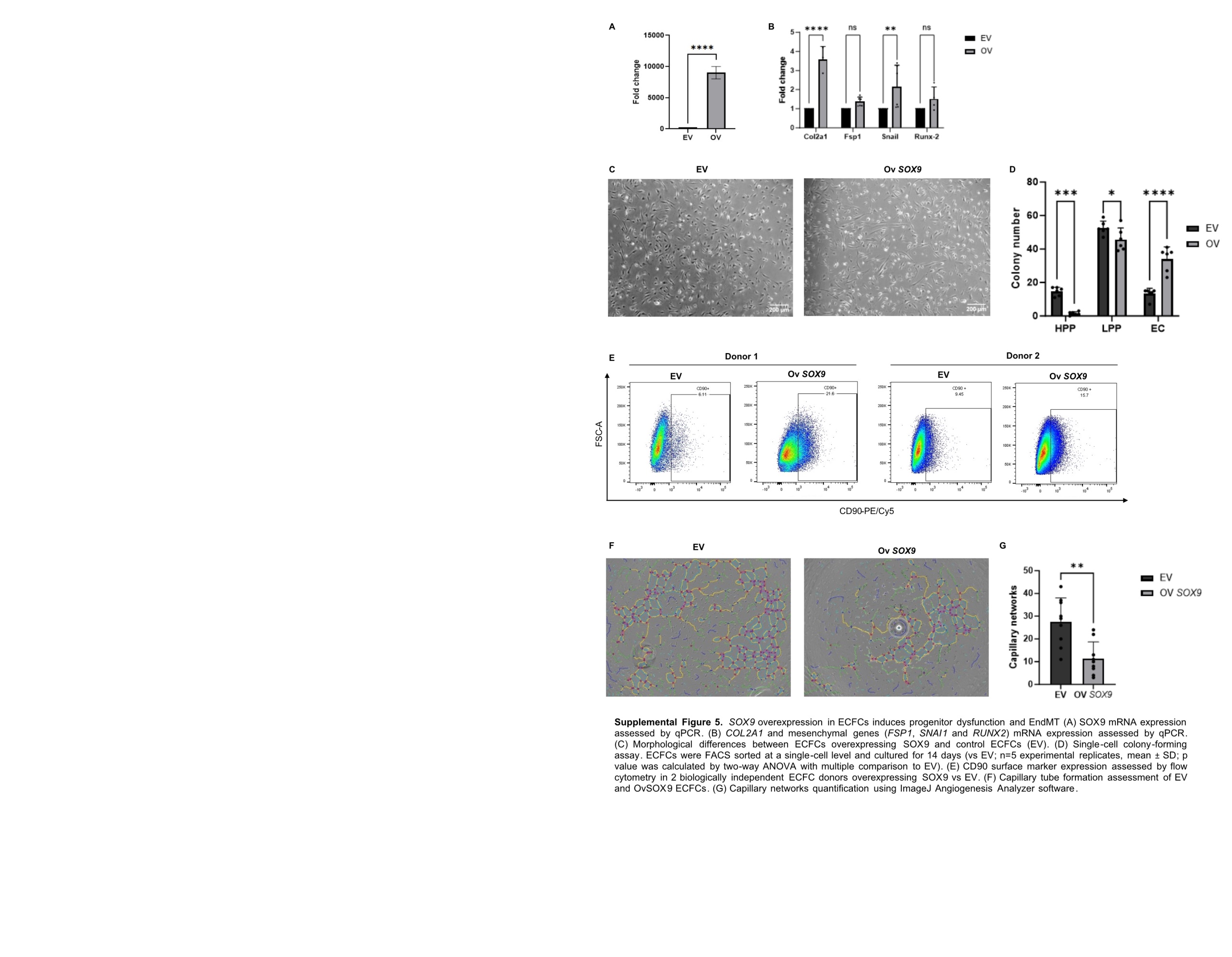



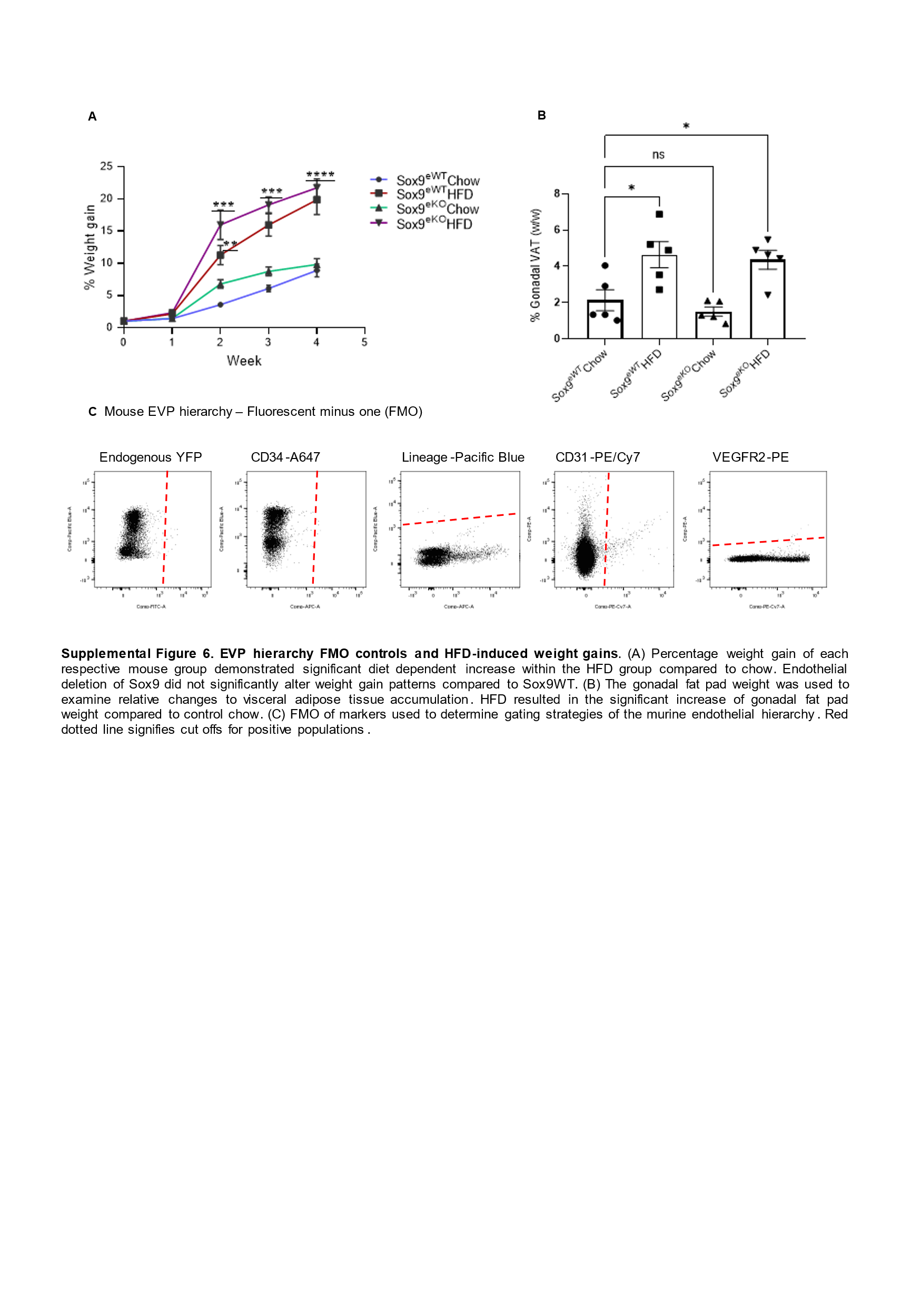


**Supplemental Methods**

**Immunofluorescence - mouse**

Dissected tissues were fixed for 30 mins at room temperature in 4% PFA. The fixative was removed with 3x washes of 1x PBS (Amresco, Solon, Ohio, USA). Tissues were subsequently infused with sucrose before cryo-embedding. For staining of specific antigens, cryo-sections were permeabilized in 0.5% Triton-x-100 (Chem Supply, Gillman, South Australia) before blocking with 20% normal goat serum. Within this study, primary antibodies used for mouse experiments included rat anti-mouse CD31 (1:100), rabbit anti-FSP1 (1:200), rabbit anti-SOX9 (1:500), and chicken anti-GFP (1:300). Excess and unbound antibody was then removed with 3x 5mins washes in a solution containing 1x PBS/0.1% Tween-20 (Amresco, Solon, Ohio, USA). Secondary antibodies conjugated with Alexa-fluor 488, 568, 647 (Invitrogen, Carlsbad, CA, USA) were used for fluorescence detection. Briefly, sections were incubated with secondary antibodies for 40mins at room temperature. Excess antibody was removed by 3x washes in PBS/0.1% Tween-20. Nuclear staining was revealed in specimens mounted with ProLong® Gold mounting media containing DAPI (Invitrogen, Carlsbad, CA, USA).

**Immunofluorescence – Human ECFCs**

ECFCs were cultured and treated on 6 mm glass coverslips coated with 1% collagen I. After the treatment period, cells were washed with PBS to remove excess media and serum, and then fixed in ice-cold 4% paraformaldehyde for 1 minute. The fixative was then removed with PBS washes. The cells were permeabilised and blocked with 0.1% TritonX-100 and 10% goat serum, 3% BSA in PBS respectively. Diluted primary antibodies were incubated overnight at 4 oC as follows: Rat anti-CD31 (#561653, BD Biosciences, CA, US, 1:100), rat anti-VE-Cadherin (1:50, BD Biosciences Cat# 560874, RRID:AB_10565967), rabbit anti-SOX9 (Millipore Cat# AB5535, RRID:AB_2239761), rabbit anti-RUNX2 (Abcam Cat# ab23981, RRID:AB_777785) and rat anti-CD90 (BioLegend Cat# 328111, RRID:AB_893441). After washing three times with PBS, diluted secondary antibody goat Alexa Fluor 488, 647 (Invitrogen, 1:500) was applied for 1 hour at room temperature. Nuclei was identified via DAPI (1:10,000) staining for 10 minutes at room temperature. Coverslips were mounted on to Superfrost™ Plus slides (Thermo Fisher Scientifics) using DAKO mounting medium (Agilent, CA, US). All images were obtained using the CSU-W1 SoRa Nikon Spinning Disk confocal microscope (Nikon, Japan). For cell counting and quantification, images were coded and analysed in a blindered manner. For quantification of transcription factor expression, at least 4 slides were evaluated per ECFC donor, with a minimum of 4 donors. Data were averaged per donor, to be used for statistical analyses.

**Microscopy**

Confocal images were acquired with either the Olympus FV3000 confocal microscope or CSU-W1 SoRa Nikon Spinning Disk microscopes using Fluoview FV3000 software v1.0 and NIS imaging software v4.5 respectively. Confocal images were acquired with a microscope equipped with Argon 561 − 10 nm DPSS and 633 nm HeNe lasers, and a 405 − 30 nm diode. Images were obtained at 20x, and 40x. Immunofluorescence vessel quantification was conducted using ImageJ v1.53 and NIS elements v4.60.00. PSR collagen quantification was conducted using Olyvia v3.2.1.

**Tissue processing and digestion**

Tissues were collected for ex vivo analyses at defined specific end time points (D5, 7, and D28). Aorta were first digested for 20 mins at 37°C in 1mg/ml collagenase I (Gibco, Life Technologies, NY, USA), 1mg/ml dispase (Gibco, Life Technologies, NY, USA), 150μg/ml DNase-I (Sigma-Aldrich, St Louis, MO, USA) before passing the suspension through a 70μM cell strainer. Cells were then washed and resuspeneded in 1x PBS 50µM EDTA 0.5% BSA solution (FACS buffer). Single cell suspensions were then used for FACS sorting or analysis by flow cytometry.

**Flow Cytometry - mouse**

Dissociated single cells in FACS buffer were then incubated with various antibody combinations for multi-parameter flow acquisition and analysis. A BD LSRFortessa™ X-20 Cell Analyser was used for sample acquisition, while unbiased data analyses were performed with FlowJo v10 software (Becton Dickinson, Franklin Lakes, NJ, USA). FACS was performed by using a FACSaria cell sorter using FACSDiva v5.0.3 with subsequent analysis performed with FlowJo v10 (Becton Dickinson). Care was taken during cell sorting to ensure only ‘singlets’ were being gated and any potential ‘doublets’ were being gated out. The following combinations of antibodies were used to assess the mouse endothelial hierarchy populations: Rat anti-mouse CD31 PE-Cy7 (1:1000) and CD34 Alexa647 (1:100) (Becton Dickinson, NJ, USA), Rat anti-mouse Lineage cocktail Pacific Blue (1:50) (Biolgend).

**Flow Cytometry – Human ECFCs**

Cultured ECFCs were dissociated from the culture flask via TrypLE™ Express Enzyme (1x) (Invitrogen) and then washed with FACS buffer. The single cell suspension in FACS buffer was then incubated with either mouse anti-human CD34 PE (MCA547GT, Bio-Rad, 1:50) or Mouse anti-human CD90 PE-Cy5 (#328111, BioLegend, 1:500). FACS acquisition was conducted on the BD BD LSRFortessa™ X-20 Cell Analyser, with unbiased data analyses performed with FlowJo v10 software. Single-stain and fluorescence-minus-one (FMO) control method was used to set voltage, compensation values and gating strategies.

**Western blot**

Cells were lysed in RIPA buffer supplemented with complete Protease Inhibitor (Sigma-Aldrich) followed by sonication. Immunoblotting of cell lysates was performed according to standard conditions. Immunoblots were labelled with following primary antibodies: rabbit anti-SOX9 (1:500, Merck Millipore, Burlington, Massachusetts, USA), rabbit anti-RUNX2 (1:500, Abcam) and mouse anti-β-actin (1:5000, Sigma-Aldrich). Primary antibodies were detected using fluorescently conjugated secondary antibodies: goat anti-rabbit IgG IRDye® 800CW and goat anti-mouse IgG IRDye® 680RN (1:2500, LI-COR Biosciences, Lincoln, Nebraska, USA). Detection and quantification of fluorescence intensity were performed using an Odyssey CLx imaging system (LI-COR Biosciences, Lincoln) and Odyssey 2.1 software or ChemiDoc MP Imaging System and ImageLab software v6.1.

**RNA Extraction, cDNA synthesis and qPCR**

RNA was extracted from FACS sorted or cultured cells using a QIAGEN mini kit (Qiagen, Valencia, CA) according to the manufacturer’s instructions. RNA quality and concentration were assessed using A260nm/A280nm spectroscopy on the Nanodrop ND-1000 (Thermo-scientific, Langenselbold, Germany). 5-100ng of RNA was used for cDNA synthesis using the Superscript III Reverse Transcription Kit (Invitrogen, Mount Waverley, Australia). To quantify relative changes in key gene expression after treatment, Real Time-Quantitative Polymerase Chain Reaction (qPCR) was conducted on cDNA using SYBR Green Master Mix reagent (Applied Biosystems, United Kingdom). Fold change of gene expression was determined using the delta delta Ct method. HPRT1 was used as housekeeper. Primer sequences are listed in Supplemental Table 2.

**RNA-sequencing**

RNA-Seq libraries were prepared using the Illumina stranded total RNA prep ligation with Ribo-Zero plus kit (Illumina, 20040529) and IDT for Illumina RNA UD Indexes (Illumina, 20040553/20040554/20040555/20040556) according to the standard manufacturer’s protocol (Illumina, Document # 1000000124514 v01). The libraries were quantified on the Perkin Elmer LabChip GX Touch with the DNA High Sensitivity Reagent kit (Perkin Elmer, CLS760672). Libraries were pooled in equimolar ratios, and the pool was quantified by qPCR using the KAPA Library Quantification Kit – Illumina/Universal (KAPA Biosystems, KK4824) in combination with the Life Technologies Viia 7 real time PCR instrument.

Sequencing was performed using the Illumina NextSeq500 (NextSeq control software v4.0.0 / Real Time Analysis v2.11.3). The library pool was diluted and denatured according to the standard NextSeq protocol (Document # 15048776 v16) and sequenced to generate single-end 76 bp reads using a 75 cycle NextSeq500/550 High Output reagent Kit v2.5 (Illumina, 20024906). After sequencing, fastq files were generated using bcl2fastq2 (v2.20.0.422), which included trimming the first cycle of the insert read due to expected low diversity. Library Preparation and Sequencing was performed at UQ Sequencing Facility based at the Institute for Molecular Bioscience (University of Queensland)

**RNA-seq Analysis**

- 1. DEG analysis and gene set enrichment analysis on bulk RNAseq data.

Read pairs from sequenced libraries were mapped to the human reference genome (GRCh38) and converted to a raw gene count table using the Rsubread tool in R ^65^After filtering out lowly expressed genes, we performed differential expression analysis across different conditions (oxLDL versus vehicle) using the standard Limma-Voom pipeline^66^.

Gene set enrichment analysis was performed using the fgsea package. Two endothelial cell signature gene sets were downloaded from the Molecular Signatures Database (MsigDB) ^67,68^and a previously published RNAseq dataset from our lab^28^.The mesenchymal cell signature gene set was obtained from the previously published RNAseq dataset from our lab^28^The endothelial-to-mesenchymal transition (EndMT) signature gene set was downloaded from a recently published RNAseq dataset^69^. Normalized enrichment score (NES) and adjusted p value were calculated for each gene set. Gene sets with high NES were relatively more enriched when compared to gene sets with low NES.

**ATAC sequencing**

The ATAC-seq protocol was adapted from previously published articles^70,71^. Briefly, 50,000 ECFCs were trypsinized and washed twice in cold PBS. Pelleted cells were lysed and then directly transposed using the TDE1 DNA Enzyme and Buffer kit (Illumina, CA, US). The transposition reaction was completed with thermomixing at 37°C for 30 minutes at 300 rpm. Transposed DNA was purified with MinElute column cleanup (Qiagen, Valencia, CA), then amplified for sequencing as previously described^72^ . Prepared libraries were purified with MinElute column clean-up (Qiagen). The ATAC-seq library pool was quantified on the Agilent Bioanalyzer with the High Sensitivity DNA kit (Agilent Technologies, 4067–4626). Sequencing was performed using the Illumina NextSeq500 (NextSeq control software v4.0.0 / Real Time Analysis v2.11.3). The library pool was diluted and denatured according to the standard NextSeq protocol (Document # 15048776 v16) and sequenced to generate paired-end 76 bp reads using a 150 cycle NextSeq500/550 Mid Output reagent Kit v2.5 (Illumina, 20024904). After sequencing, fastq files were generated using bcl2fastq2 (v2.20.0.422).

Sequencing was performed at UQ Sequencing Facility based at the Institute for Molecular Bioscience (University of Queensland).

**ATAC-seq Analysis**

Raw paired-end sequencing reads were aligned to the hg19 reference genome using Bowtie2^73^(version 2.2.9), after removing sequencing adapters with Cutadapt (version 1.13). Samtools (version 1.9) and Picard were used to filter duplicates, remove reads with mapping quality < 30, unmapped reads, reads aligning to the mitochondrial chromosome and unplaced contigs. Quality of library enrichment was evaluated using ENCODE metrics, including Transcription Start Site signal > 10, and sample concordance examined using clustering analysis. Peaks were called for each sample using MACS2 (version 2.1.2), with parameters“-p 0.01–-nomodel–-shift 0–-extsize 14”. Differentially accessible regions between treated and untreated samples were called using DiffBind version 2.14.0, where treatment-dependent peaks were selected as those exhibiting a log fold change > 1. Over-represented transcription factor binding motifs within ATAC-seq peaks were identified using HOMER version 4.8. We also employed a permutation-based strategy using ENCODE dNase-seq footprinting data^74^. Briefly, motifs for selected TFs (SOX9, GLI1, ZEB1 and CTCF) were intersected with treatment-dependent open chromatin peaks. The observed number of intersected motifs was compared to null background sets by computing the overlap in 100 iterations of randomly shuffled, equal sized sets of non-treatment associated peaks. Over-represented pathways based were detected using the ReactomePA Bioconductor package^75^, by assigning differential peaks to genes within 5 kb.

**Supplementary Table 1 - Antibodies**

**Table 1.1 – Mouse endovascular progenitor hierarchy panel**

| **Protein Target** | **Manufacturer** | **RRID** | **Dilution** | **Conjugated**  **Fluorophore** |
| --- | --- | --- | --- | --- |
| Endogenous YFP | Endogenous | N/A | N/A | YFP |
| Rat-anti-mouse CD31 | BD Biosciences, 561410 | AB_10612003 | 1:600 | PE-Cy7 |
| Rat-anti-mouse CD34 | BD Biosciences, 560230 | AB_1645200 | 1:150 | AlexaFluor647 |
| Rat-anti-mouse Lineage Cocktail (Gr-1, Ter-119, CD3, CD11b, CD45) | BioLegend,  133310 | AB_11150779 | 1:50 | Pacific Blue |
| 7’AAD viability stain | BioLegend  420404 | N/A | 1:20 | PE-Cy5 |

**Table 1.2 – Mouse aortic myeloid panel**

| **Protein Target** | **Manufacturer** | **Dilution** | **RRID** | **Conjugated Fluorophore** |
| --- | --- | --- | --- | --- |
| Rat-anti-mouse CD45 | BD Biosciences, 563410 | 1:800 | AB_2738189 | BV650 |
| Rat-anti-mouse CD64 | BD Biosciences, 652872 | 1:200 |  | BV421 |
| Rat-anti-mouse MHCII | BioLegend,  109905 | 1:100 | AB_313454 | FITC |
| Rat-anti-mouse CD11b | BioLegend,  101227 | 1:200 | AB_893233 | PerCP/Cy5.5 |
| Hamster-anti-mouse CD11c | BioLegend,  117317 | 1:200 | AB_493569 | PE/Cy7 |
| Rat-anti-mouse Ly6g | BioLegend,  127607 | 1:200 | AB_1186104 | PE |
| Rat-anti-mouse Ly6c | BioLegend,  128025 | 1:200 | AB_10643867 | APC |
| 7’AAD viability stain | BioLegend  420404 | 1:20 | N/A | PE-Cy5 |

**Table 1.3 – Human ECFC EndMT**

| **Protein Target** | **Manufacturer** | **Dilution** | **RRID** | **Conjugated Fluorophore** |
| --- | --- | --- | --- | --- |
| Mouse-anti-human CD34 | Bio-Rad Laboratories,  MCA547GT | 1:50 | AB_2063000 | PE |
| Mouse-anti-human CD31 | BD Biosciences, 561653 | 1:200 | AB_10896326 | BV421 |
| Mouse-anti-human CD90 | BD Biosciences,  555597 | 1:1000 | AB_395971 | PE-Cy5 |
| FVS700 | BD Biosciences,  564997 | 1:8000 | AB_2869637 | AlexaFluor700 |

**Supplementary Table 2 – qPCR Primers**

| **Target** | **Forward Primer** | **Reverse Primer** |
| --- | --- | --- |
| *HPRT* | CCTGGCGTCGTGATTAGTGAT | AGACGTTCAGTCCTGTCCATAA |
| *SOX9* | AGCGAACGCACATCAAGAC | CTGTAGGCGATCTGTTGGGG |
| *HEY1* | TGGAGAGGCGCCGCTGTAGTTA | CAAGGGCGTGCGCGTCAAAGTA |
| *FSP-1* | GCCCTGGATGTGATGGTGT | TCGTTGTCCCTGTTGCTGTC |
| *RUNX2* | GTTATGAAAAACCAAGTAGCAAGG | GTAATCTGACTCTGTCCTTGTGGAT |
| *SNAI* | CTTTTTCTTGCCCTCACTGC | ACAGCAGCCAGATTCCTCAT |
| *CDH5* | CAGCCCAAAGTGTGTGAGAA | TGTGATGTTGGCCGTGTTAT |
